# Fixed-time angle of attack constrained control for aircraft considering dynamic icing process

**DOI:** 10.1038/s41598-023-50038-y

**Published:** 2024-03-04

**Authors:** Zehong Dong, Xingya Da, Yinghui Li, Zhe Li, Like Xie

**Affiliations:** 1 China Aerodynamics Research and Development Center, High Speed Aerodynamics Institute, Mianyang, 621000 China; 2 Aviation Engineering School, Air Force Engineering University, Xi’an, 710038 China; 3 China Aerodynamics Research and Development Center, Low Speed Aerodynamics Institute, Mianyang, 621000 China

**Keywords:** Applied mathematics, Information technology, Aerospace engineering, Electrical and electronic engineering

## Abstract

Aircraft icing deteriorates aerodynamic performance and reduces stall angle of attack, the fast convergence rate of tracking error is required to stabilize the aircraft when aircraft icing occurs. The state-of-the-art control methods for icing aircraft mostly assume that the icing of aircraft is instantaneous. Aiming at these issues, a fixed-time angle of attack-constrained control strategy is designed considering dynamic icing process. In order to explore the variation of aerodynamic coefficients in the process of dynamic icing, an ice wind tunnel experiment is implemented, and the relationship between lift coefficient, drag coefficient and pitching moment coefficient with angle of attack and icing intensity is obtained by fitting method. In order to prevent the stalling problem caused by the decrease of the stalling angle of attack in the process of dynamic icing, a method to determine the stalling angle of attack based on deep neural network is proposed. Considering the asymmetric and time-varying angle of attack constraint, a fixed-time convergent angle of attack-constrained robust control method is designed. The ice wind tunnel experiment shows the process of dynamic icing of the airfoil, and the simulation results verify the effectiveness of the proposed control method.

## Introduction

Aircraft icing not only increases the weight of aircraft, but also destroys the flow field around the surface of airframe and changes the dynamic characteristics of aircraft, resulting in reduced lift and increased drag, which in turn reduces the stall angle of attack and increases the stall speed of aircraft, bringing great hidden dangers to flight safety^1,2^. Although the research on aircraft icing has lasted for decades, and rich experience has been accumulated in theory and practice, flight accidents caused by aircraft icing still continue to occur^3^. Aircraft icing affects the maneuverability and stability of aircraft, and many flight accidents are caused by the fact that the flight control system is not robust enough to aircraft icing^4^. Therefore, it is urgent to design a robust control system considering the adverse effects of aircraft icing.

In recent years, a lot of effort about aircraft icing problem has been made, including ice wind tunnel experiment^5^, dynamic inverse control^6^, stability region estimation^7^, icing protection system^8^, etc. However, the above literature only focuses on the robustness of control system to aircraft icing, ignoring the convergence rate of tracking errors. The operating stability of aircraft deteriorates when aircraft icing occurs. If the tracking errors cannot converge quickly during maneuvering, the system instability is easy to be induced. In order to improve the convergence rate of tracking errors, the finite-time stability control is applied to the design of tracking controller. On the basis of the exponential convergence controller, a fractional power term about tracking error is added (the power index is between 0 and 1)^9^. Although the finite-time control improves the convergence rate, the convergence time is related to the initial state of the system. When the initial state cannot be accurately measured, it is difficult to calculate the convergence time of the system. To solve this issue, the fixed-time tracking controller is proposed. On the basis of the finite-time controller, it adds a fractional power term about the tracking error (the power index is greater than 1), so that the convergence time of the tracking errors does not depend on the initial value of the system^10–13^. Fixed-time tracking control has the advantage of making the system fast and stable, which has important theoretical and application value in the design of flight control system^14,15^. However, there is little literature on the fixed-time robust control for icing aircraft. Therefore, how to design a fixed-time robust tracking controller for icing aircraft is an urgent problem.

Noting that the stall angle of attack will decrease when the aircraft icing happens. If the control law of clean aircraft is still used, it is easy to cause the stall problem. The constrained control of the angle of attack for icing aircraft is a problem that needs to be solved urgently^16^. It is worth mentioning that aircraft icing is a dynamic process. According to the ice wind tunnel experiment, the stall angle of attack decreases with the increase of icing intensity^17^. In the process of designing the control law, the constraint of angle of attack should also change with icing intensity, and the constraint of angle of attack is asymmetric and time-varying^18^. Therefore, it is of great significance to explore the change rule of stall angle of attack with icing intensity considering dynamic icing process. In order to meet the constraint of angle of attack^19^, propose a angle of attack-constrained controller based on the barrier function, but only the time-invarying and symmetric constraint of angle of attack is taken into account, where the smallest constraint of angle of attack is adopted within the full flight envelope and such consideration is relatively conservative. An integrated design method is proposed for guidance and control of flight vehicle considering constraints based on barrier function^20^. The variation of flight environment is considered and an adaptive control method considering asymmetric and time-varying constraint of angle of attack is proposed^21^. However, the convergence rate of tracking errors in the above methods is not taken into full consideration.

In order to solve the above problems, this paper proposes a fixed-time angle of attack-constrained control strategy for aircraft considering dynamic icing process. The main innovations are as follows:

- The dynamic process of airfoil icing in flight is simulated in the ice wind tunnel. The change rule of aerodynamic coefficients with respect to angle of attack and icing intensity is obtained by mathematical analysis based on experimental data.
- The relationship between stall angle of attack and icing intensity is given by applying the deep neural network.
- Considering the dynamic icing process, a fixed-time angle of attack-constrained robust controller is designed to make the system quickly stable and ensure that the angle of attack does not exceed the stall angle of attack. Compared with the state-of-the-art fixed-time angle of attack-constrained control methods, the error transformation is not required via the proposed methodology, which provides a means of limiting the angle of attack directly.

The remainder of this article is organized as follows. In “Icing aircraft dynamic model and preliminaries” section, the aerodynamic coefficients under dynamic icing condition and the change rule of stall angle of attack with icing intensity, and preliminaries are provided. Section “Fixed-time angle of attack-constrained robust controller design” section gives the fixed-time angle of attack-constrained robust control method. The closed-loop stability analysis is given in “Stability analysis” section. In “Simulation results” section, the comparative simulation results are given. Finally, “Conclusion” section concludes the work.

## Icing aircraft dynamic model and preliminaries

### Icing aircraft dynamic model

The icing aircraft dynamic model is based on the NASA’s Generic Transport Model (GTM) in aviation, of which longitudinal dynamic model can be expressed as

1 $$\begin{aligned} \left\{ {\begin{array}{*{20}{l}} \dot{V} = \frac{1}{m}\left( {{F_x}\cos \alpha + {F_z}\sin \alpha } \right) + {d_V}, \\ \dot{h} = V\sin \left( \gamma \right) + {d_h}, \\ {\dot{\gamma }} = \frac{1}{{m{V}}}\left( {{F_x}\sin \alpha - {F_z}\cos \alpha } \right) + {d_\gamma }, \\ {\dot{\alpha }} = Q + \frac{1}{{m{V}}}\left( { - {F_x}\sin \alpha + {F_z}\cos \alpha } \right) + {d_\alpha }, \\ \dot{Q} = \frac{{{M_y}}}{{{J_y}}} + {d_Q}, \end{array}} \right. \end{aligned}$$

where

2 $$\begin{aligned} \left\{ {\begin{array}{*{20}{l}} {F_x} = {\bar{q}}{S_{ref}}{C_x} + 2{T_x} - mg\sin \theta , \\ {F_z} = {\bar{q}}{S_{ref}}{C_z} + 2{T_z} + mg\cos \theta , \\ {M_y} = {\bar{q}}{S_{ref}}{\bar{c}}{C_m}, \end{array}} \right. \end{aligned}$$

where *V* is the velocity; *m* is the mass; $\theta$ is the pitch angle; $\alpha$ is the angle of attack; *Q* is the pitch rate; ${\bar{c}}$ is the mean aerodynamic chord; $S_{ref}$ is the reference wing surface area; $J_y$ is the moment inertias along aircraft *y* axis; *g* is the acceleration of gravity; ${\bar{q}} = {\textstyle {1 \over 2}}\rho V^2$ is the dynamic pressure with $\rho$ being the atmospheric density. The main structure parameters can be obtained by consulting full-scale model of NASA’s GTM^18^ and the detailed polynomial model parameters of axial force coefficient $C_x$, normal force coefficient $C_z$ and pitching moment coefficient $C_m$ can be found in paper which is obtained from flight test^22^, where they are polynomial functions of $\alpha$, *q* and the elevator deflection $\delta _e$; ${T_x}$ and ${T_z}$ are the aerodynamic forces along the *x* axis and *z* axis, respectively.

The icing factor model developed by Bragg’s team is used to determine the aerodynamic coefficient model of icing aircraft, which is expressed as^18^

3 $$\begin{aligned} {C_{A,iced}}{{ = }}\left( {{{1 + }}\eta {f_{ice}}} \right) {C_A}, \end{aligned}$$

where ${C_A}$ and $C_{A,iced}$ are the aerodynamic derivative values of aircraft before and after icing, respectively; $f_{ice}$ is the icing coefficient, which reflects the sensitivity of ${C_A}$ to aircraft icing.

**Figure 1:**
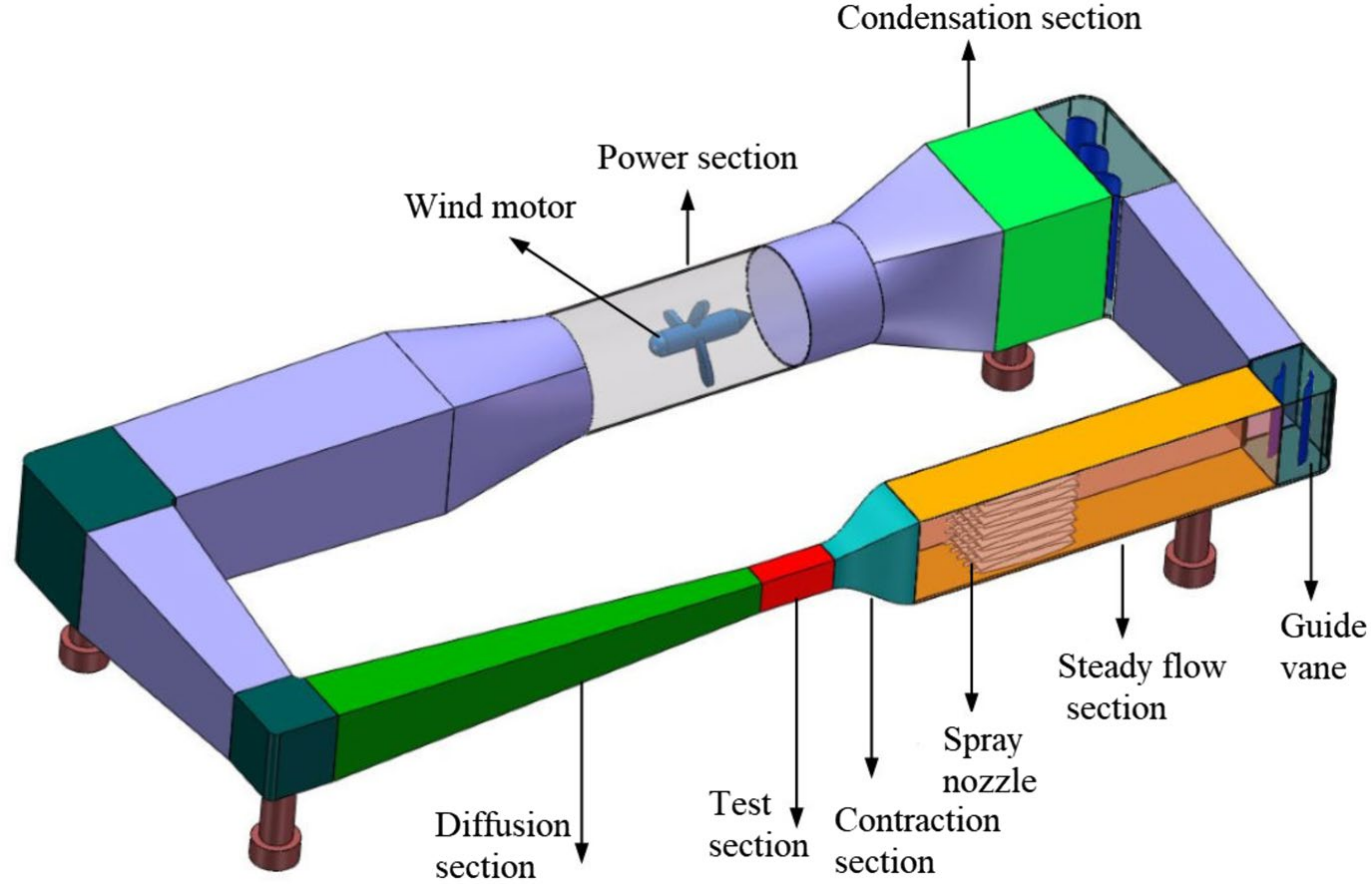
Schematic diagram of icing wind tunnel structure.

**Figure 2:**
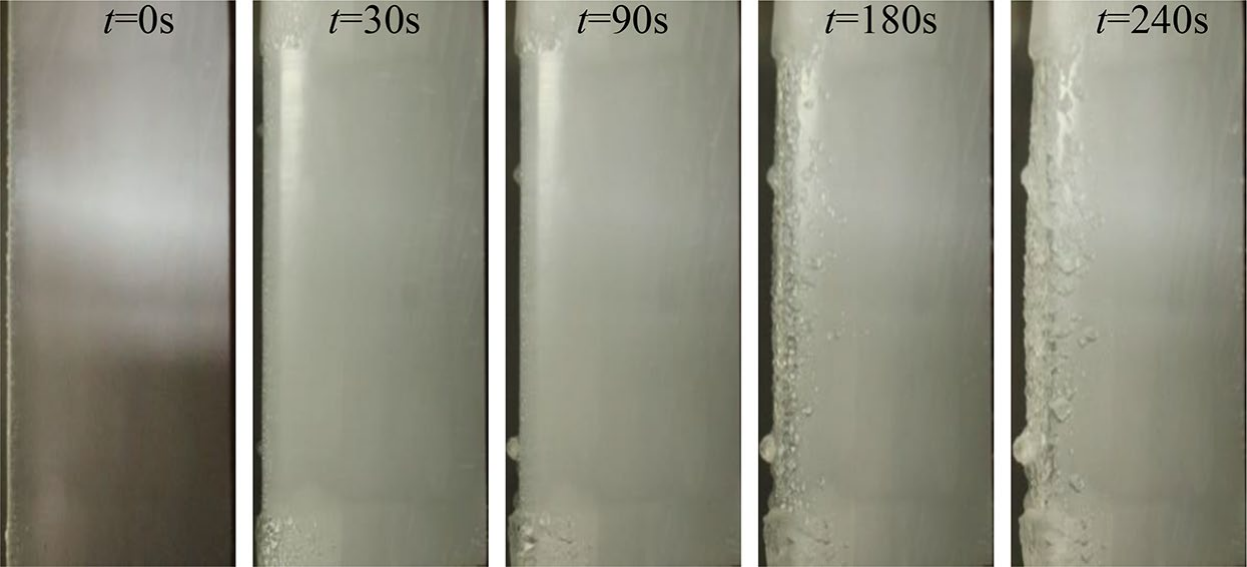
The dynamic icing process.

**Figure 3:**
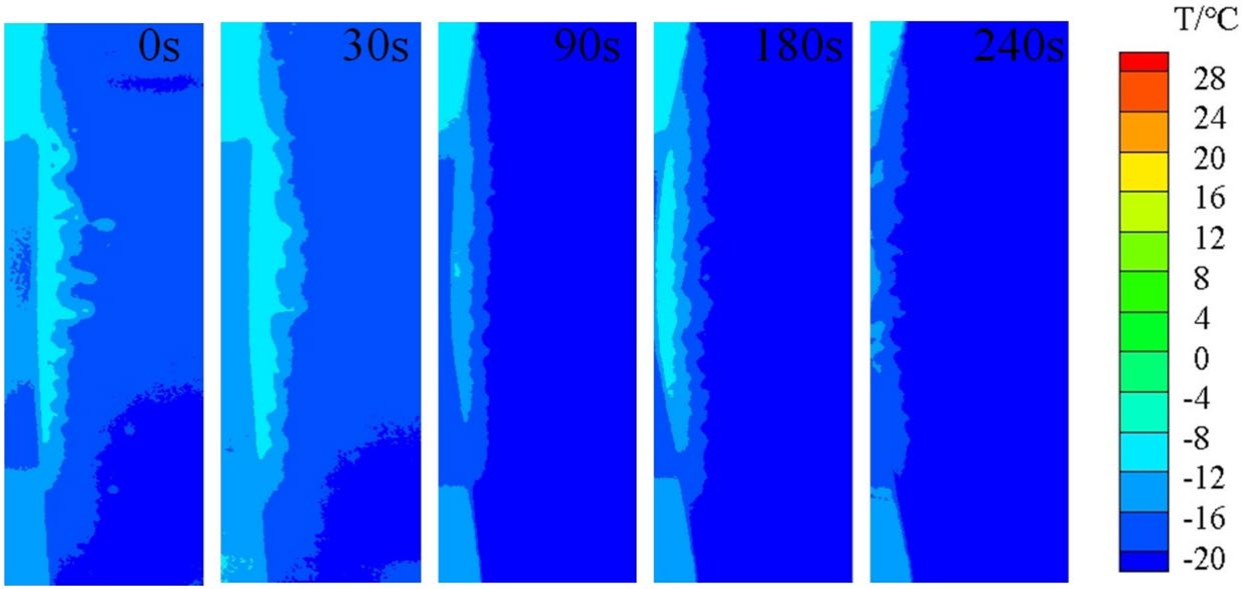
The temperature distribution cloud map of the airfoil surface.

Aircraft icing is a dynamic process, rather than an instant completion. According to the research results of NASA^23,24^, it takes about 4 min to reach the degree of severe aircraft icing. An ice wind tunnel experiment was carried out at the China Aerodynamics Research and Development Center to simulate the dynamic icing process in flight. As shown in Fig. 1, the icing wind tunnel has a closed-loop structure, including the power section, condensation section, steady flow section, contraction section, test section and diffusion section. The test section of icing wind tunnel is rectangular, 0.65 m long, 0.3 m wide and 0.2 m high. The maximum wind speed is 170 m/s. The median volume droplet diameter of the wind tunnel can be adjusted according to the ratio of water supply and gas supply pressure. Within the range of 20–50${\upmu } \textrm{m}$, the liquid water content can be adjusted to 0.5g/m$^3$ or 1g/m$^3$ by changing the number of nozzles. The adjustable temperature range of the test section is − 40 $^ \circ$C to normal temperature. Figure 2 shows the icing situation of the wing in 0–4 min. The airfoil surface temperature distribution at different time is shown in Fig. 3, indicating that the temperature of airfoil surface is lower than 0 $^ \circ$C in the whole process and the temperature gets colder and colder as the icing intensity increases.

**Figure 4:**
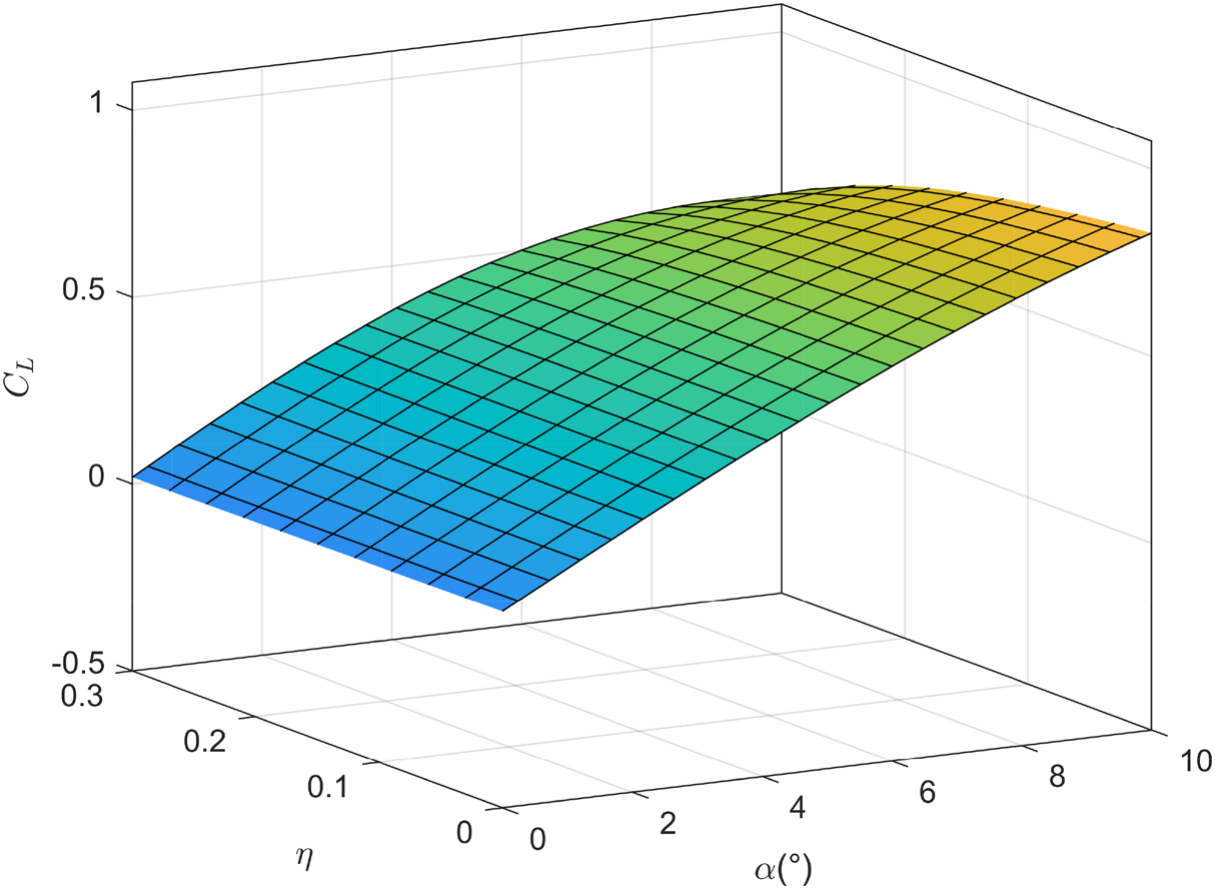
The variation of lift coefficient with angle of attack and icing degree.

**Figure 5:**
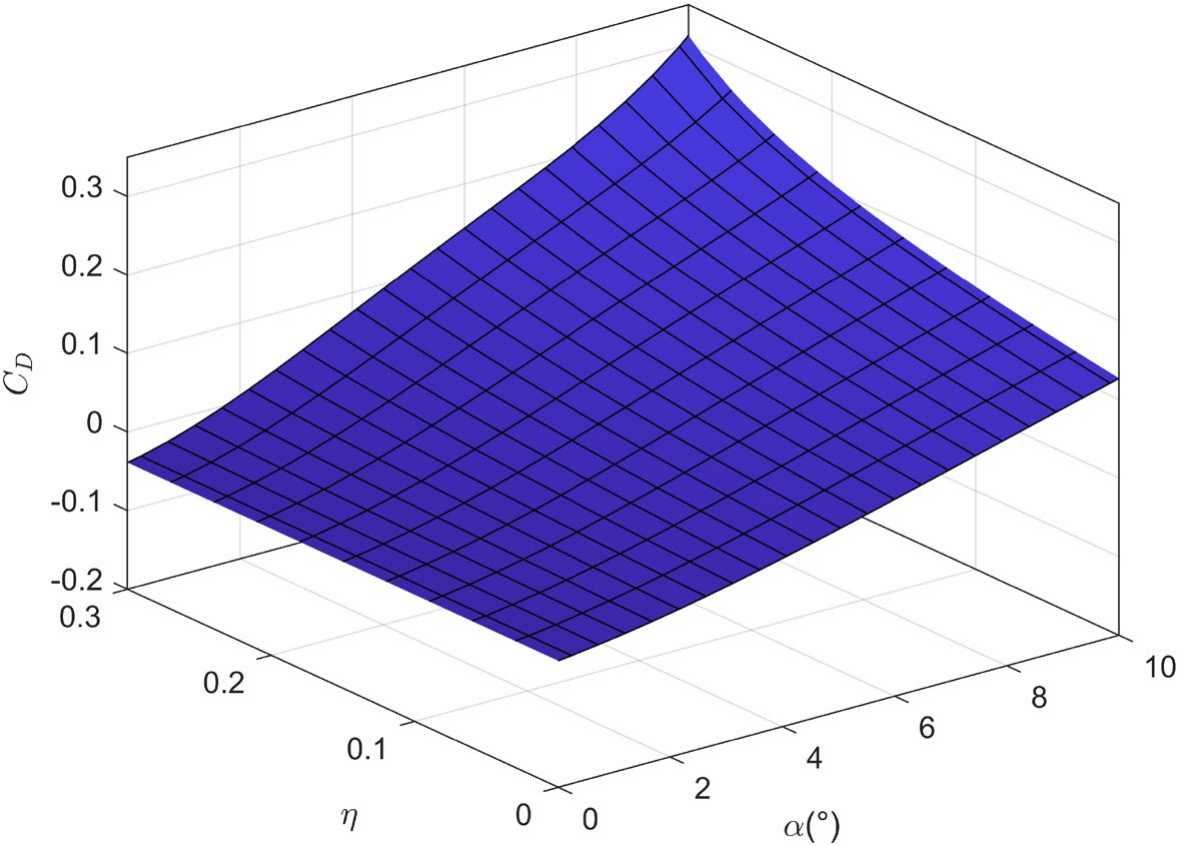
The variation of drag coefficient with angle of attack and icing degree.

**Figure 6:**
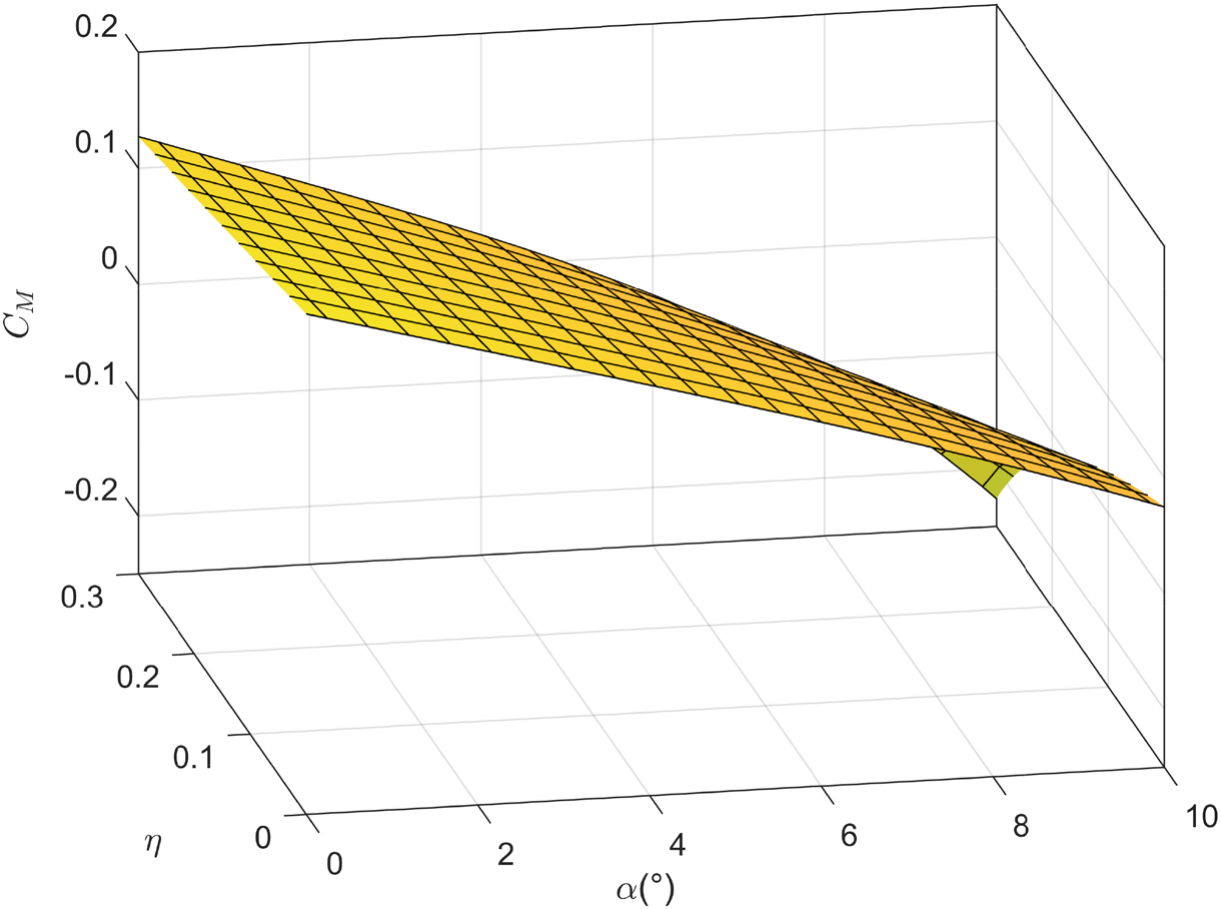
The variation of pitch moment coefficient with angle of attack and icing degree.

It is worth noting that the experimental results are noisy in general, in order to facilitate the design of the controller, the mathematical fitting method is used to fit the lift coefficient, drag coefficient and pitching moment coefficient obtained from the experiment into a smooth surface, as shown in Figs. 4, 5 and 6. It can be seen that with the increase of icing intensity, the lift coefficient decreases, the drag coefficient increases, and the nonlinear characteristic of pitching moment intensifies. In order to truly simulate the impact of aircraft icing on flight safety, the linear function is adopted to simulate the dynamic process of aircraft icing, namely:

4 $$\begin{aligned} {C_{A,iced}}\left( t \right) {{ = }}\left\{ {\begin{array}{*{20}{l}} {{C_A},}&{}{t \le {t_0}},\\ {{C_A} + \frac{{{C_{A,iced}} - {C_A}}}{{{t_{max}}}}\left( {{{1 + }}\eta {f_{ice}}} \right) {C_A},}&{}{{t_0} \le t \le {t_{max}}},\\ {{C_{A,iced}},}&{}{t > {t_{max}}}, \end{array}} \right. \end{aligned}$$

where $t_0$ is the initial time of aircraft icing; $t_{max}$ is the completion time of aircraft icing.

What’s more, the aerodynamic data can be generated from computer experiments, such as CFD^25^.

### Analysis of aerodynamic characteristics of dynamic icing process

**Figure 7:**
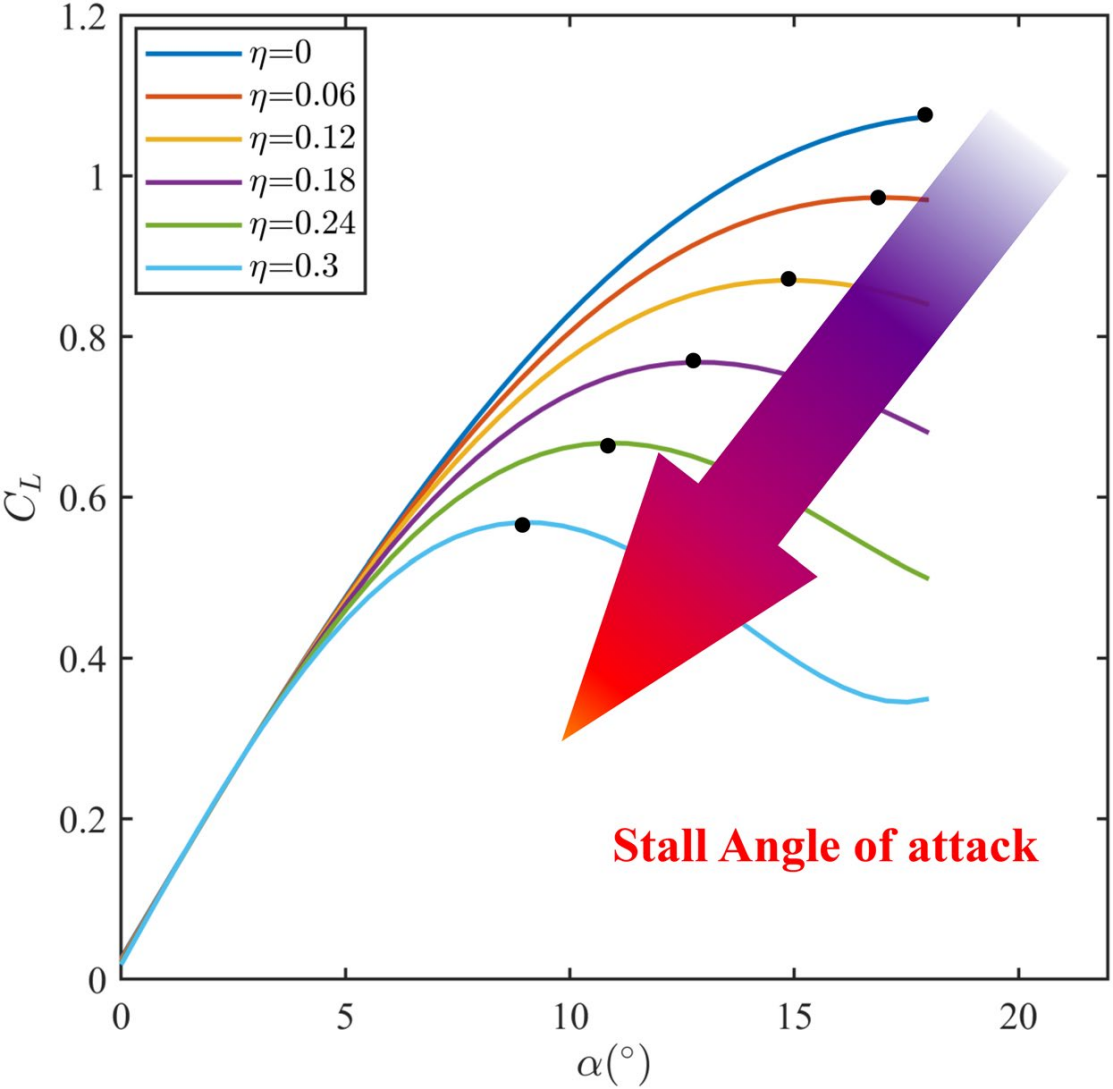
The variation of lift coefficient curve with increasing ice degree.

**Figure 8:**
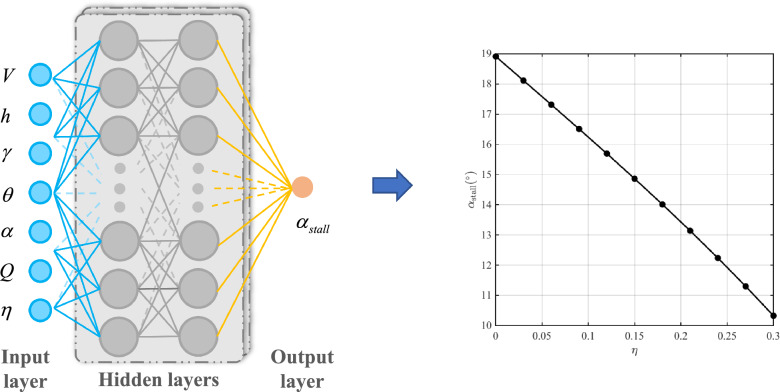
The structure diagram of deep neural network to determine stall angle of attack.

With the increase of icing intensity, the lift coefficient decreases gradually and the stall angle of attack decreases, as shown in Fig. 7. The stall angle of attack is affected by multiple factors, which are coupled to each other. That makes the change rule of stall angle of attack intricate. In order to obtain the stall angle of attack based on the flight states and icing intensity in real time, the deep neural network is applied to characterize the relationship between the flight states, icing intensity and stall angle of attack. The deep neural network is trained offline and used online in flight simulation. The input vector of deep neural network is ${\left[ {V,h,\gamma ,\theta ,\alpha ,Q,\eta } \right] ^{\textrm{T}}}$, whose size is 600$\times$7, and the output is stall angle of attack. The fully connected multiple layer perceptron is employed and the ReLU activation function is utilized. The optimizer of the deep neural network is selected as SGD. The learning rate is set to 0.0001. The number of iterations is set to 1000000. The deep neural network contains two hidden layers with 128 and 256 nodes, respectively. The structure diagram of deep neural network to determine stall angle of attack is shown in Fig. 8.

### Model decomposition

In this paper, the backstepping method is used to design the controller. In order to facilitate the design of the controller, It assumes that $\sin \gamma \approx \gamma , \cos \gamma \approx 1$. Considering the external disturbance and aerodynamic parameter perturbation, the aircraft dynamic model are decomposed into velocity subsystem and altitude subsystem, which are respectively expressed as

5 $$\begin{aligned}{} & {} \dot{V} = {F_V} + {g_V}\Phi + {d_V} \end{aligned}$$

6 $$\begin{aligned}{} & {} \left\{ \begin{array}{*{20}{l}} {\dot{h} = V\gamma + {d_h}},\\ {{\dot{\gamma }} = {F_\gamma } + \alpha + {d_\gamma }},\\ {{\dot{\alpha }} = {F_\alpha } + Q + {d_\alpha }},\\ {\dot{Q} = {F_Q} + {g_Q}{\delta _{e}} + {d_Q}}, \end{array}\right. \end{aligned}$$

where

7 $$\begin{aligned} \left\{ {\begin{array}{*{20}{l}} {F_V} = \frac{{\cos \alpha }}{m}\left( {{\bar{q}}{S_{ref}}{C_{x,iced}\left( t \right) } - mg\sin \theta } \right) \\ \; \; \; \; \; \; + \frac{{\sin \alpha }}{m}\left( {{\bar{q}}{S_{ref}}{C_{z,iced}\left( t \right) } + mg\sin \theta } \right) ,\\ {g_V} = \frac{{2\cos \alpha }}{m}T_x^\Phi + \frac{{2\sin \alpha }}{m}T_z^\Phi = \frac{2}{m}C_T^\Phi \left( \alpha \right) ,\\ {{F_\gamma } = \frac{{{F_x}\sin \alpha - {F_z}\cos \alpha }}{{mV}} - \alpha },\\ {F_\alpha } = \frac{{ - {F_x}\sin \alpha + {F_z}\cos \alpha }}{{mV}},\\ {F_Q} = \frac{{{\bar{q}}{S_{ref}}{\bar{cC}}_{M,iced}^\alpha \left( t \right) }}{{{J_y}}},\\ {g_Q} = \frac{{{\bar{q}}{S_{ref}}{\bar{cC}}_{M,iced}^{{\delta _e}}\left( t \right) }}{{{J_y}}}, \end{array}} \right. \end{aligned}$$

where $T_x^\Phi$ and $T_z^\Phi$ represent the component of engine thrust along the x axis and z axis, respectively; $C_T^\Phi \left( \alpha \right)$ represents the thrust coefficient with respect to angle of attack; ${C_M^\alpha }$ denotes the pitch moment coefficient with respect to angle of attack; ${{C_M^{{\delta _e}}}}$ denotes the pitch moment coefficient with respect to elevator deflection.

#### Assumption 1

There exist unknown positive constants ${\bar{d}}_i$ such that $d_i \le {\bar{d}}_i$ ($i \in V,\gamma ,\alpha ,Q$). The values of functions $F_V, F_\gamma , F_\alpha , F_Q$ can be calculated based on the nominal aerodynamic coefficients, $g_V>0$ and $g_Q>0$ are known for the control design.

#### Assumption 2

The reference trajectories $V_{ref}$, $h_{ref}$ and their derivatives $\dot{V}_{ref}$, $\dot{h}_{ref}$ are bounded and available, and there exists a known compact set ${\Omega _0}$ such that

8 $$\begin{aligned} {\Omega _0} = \left\{ {{{\left[ {{V_{ref}},{h_{ref}},{{\dot{V}}_{ref}},{{\dot{h}}_{ref}}} \right] }^{\textrm{T}}}|V_{ref}^2 + h_{ref}^2 + \dot{V}_{ref}^2 + \dot{h}_{ref}^2 \le {B_0}} \right\} , \end{aligned}$$

with $B_0$ being a known positive constant.

#### Remark 1

The coefficient uncertainties and external disturbances are integrated into the lumped disturbances. As a result, the nominal values of $F_V, F_\gamma , F_\alpha , F_Q, g_V, g_Q>0$ are treated as known functions. Assumption 2 is a common condition required in adaptive control literature^26^. Hence, Assumptions 1–2 are reasonable.

### Preliminaries

#### Lemma 1

^27^: For nonlinear system:

9 $$\begin{aligned} \dot{\varvec{x}} \left( t \right) = f\left( {\varvec{x}\left( t \right) } \right) ,\varvec{x}\left( 0 \right) = {x_0}, \end{aligned}$$

if there exist positive constants *a*, *b*, $p>1$, $0<q<1$, $0< \eta < \infty$ such that

10 $$\begin{aligned} V\left( \varvec{x} \right) \le - a{V^p}\left( \varvec{x} \right) - b{V^q}\left( \varvec{x} \right) + \eta , \end{aligned}$$

then the system is called to be practical fixed-time stability within

11 $$\begin{aligned} {T_s} \le {T_{\max }}: = \frac{1}{{a\phi \left( {p - 1} \right) }} + \frac{1}{{b\phi \left( {1 - q} \right) }}., \end{aligned}$$

where $0< \phi < 1$ and the solution of the system will converge to the following compact set

12 $$\begin{aligned} x \in \left\{ {V\left( x \right) \le \min \left\{ {{{\left( {\frac{\eta }{{\left( {1 - \phi } \right) a}}} \right) }^{\frac{1}{p}}},{{\left( {\frac{\eta }{{\left( {1 - \phi } \right) b}}} \right) }^{\frac{1}{q}}}} \right\} } \right\} . \end{aligned}$$

#### Lemma 2

^28^: For any $a \ge 0$, $b>0$, $c>0$, the following inequality holds

13 $$\begin{aligned} {a^c}\left( {b - a} \right) \le \frac{1}{{1 + c}}\left( {{b^{1 + c}} - {a^{1 + c}}} \right) . \end{aligned}$$

#### Lemma 3

^28^: For any $a>0$, $b \le a$, $c>1$, the following inequality holds

14 $$\begin{aligned} {\left( {a - b} \right) ^c} \ge {b^c} - {a^c}. \end{aligned}$$

#### Lemma 4

^29^: For the system (9), considering the integral-type Lyapunov function candidate

15 $$\begin{aligned} {V_i} = \int _0^{{z_i}} {\frac{{\sigma k_i^2\left( t \right) }}{{k_i^2\left( t \right) - \left( {\sigma + {\alpha _{i - 1}}} \right) }}} d\sigma ,\;\;i = 1,2, \ldots ,n, \end{aligned}$$

where ${z_i} = {x_i} - {\alpha _{i - 1}}, {\alpha _0}: = {y_d},{\alpha _1},{\alpha _2}, \ldots ,{\alpha _{n - 1}}$ are continuously differentiable functions satisfying $|{{\alpha _i}} |\le {A_i} < {k_i\left( t \right) }$ for positive constants $A_i$. For $|{{x_i}} |< {k_i\left( t \right) }, \forall t\ge 0$, the following inequation holds

16 $$\begin{aligned} {V_i} \le \frac{{{z^2_i}k_i^2\left( t \right) }}{{k_i^2\left( t \right) - x_i^2}}. \end{aligned}$$

#### Lemma 5

^30^: For any $\sigma > 0$, $\varsigma \in {\textbf {R}}$, there exists constant $\kappa = 0.2785$ such that

17 $$\begin{aligned} 0 \le |\varsigma |- \varsigma \tanh \left( {\frac{\varsigma }{\sigma }} \right) \le \kappa \sigma . \end{aligned}$$

The control objective is developing a fixed-time robust controller considering the constraint of angle of attack, achieving fixed-time stability of the close-loop system and keeping the angle of attack always being within a reasonable range during the dynamic icing process.

## Fixed-time angle of attack-constrained robust controller design

**Figure 9:**
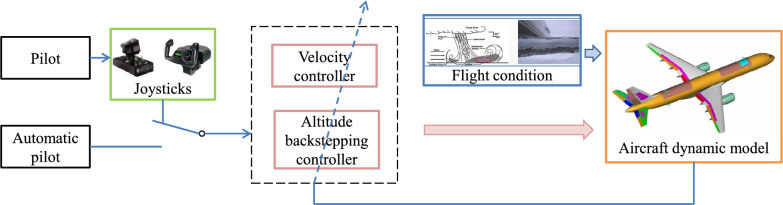
The block diagram of the angle of attack-constrained controller.

The block diagram of the angle of attack-constrained controller considering dynamic icing process is shown as Fig. 9.

### Velocity controller design

Define the tracking error of velocity as

18 $$\begin{aligned} e_V=V - V_{ref}, \end{aligned}$$

Taking the time derivative of $e_V$ and substituting it into (5), one has

19 $$\begin{aligned} \dot{e}_V= {F_V} + {g_V}\Phi + {d_V} - \dot{V}_{ref} , \end{aligned}$$

Define ${\varepsilon _V}$ as the absolute upper bound of ${F_V} + {d_V} - {\dot{V}_{ref}}$, the value of which is unknown. In order to enhance the robustness of the system, ${{\hat{\varepsilon }} _V}$ is used to estimate the value of ${\varepsilon _V}$ with the estimation error being ${{\tilde{\varepsilon }} _V} = {\varepsilon _V} - {{\hat{\varepsilon }} _V}$. The velocity controller is designed as

20 $$\begin{aligned} \Phi = \frac{1}{{{g_V}}}\left[ { - {k_{V1}}{e_V} - {k_{V2}}{\mathop {{\textrm{sg}}}\nolimits } \left( {e_V^p} \right) - {k_{V3}}{\hbar _V} - {{{\hat{\varepsilon }} }_V}\tanh \left( {\frac{{{e_V}}}{{{\sigma _V}}}} \right) } \right] , \end{aligned}$$

where $p > 1, k_{V1}, k_{V2}, k_{V3}, {\sigma _V}$ are the positve parameters to be designed. ${\mathop {{\textrm{sg}}}\nolimits } \left( {{ \bullet ^q}} \right) ={\textrm{sg}}\left( \bullet \right) {|\bullet |^q}$, where ${\textrm{sg}}\left( \bullet \right)$ is the sign function. The switching function ${\hbar _V}$ is designed as

21 $$\begin{aligned} {\hbar _V} = \left\{ {\begin{array}{*{20}{l}} {{\mathop {\textrm{sg}}\nolimits } \left( {e_V^q} \right) ,}&{}{{\textrm{if}} |{{e_V}} |> {\tau _V},}\\ {\frac{{3 - q}}{2}\tau _V^{q - 1}{e_V} + \frac{{q - 1}}{2}\tau _V^{q - 3}e_V^3,}&{}{\textrm{others}}, \end{array}} \right. \end{aligned}$$

where $0< q < 1, {\tau _V} > 0$ are the parameters to be designed.

Design the adaptive law as

22 $$\begin{aligned} {\dot{{\hat{\varepsilon }}} _V} = {e_V}\tanh \left( {\frac{{{e_V}}}{{{\sigma _V}}}} \right) - {l_{V1}}{{\hat{\varepsilon }} _V} - {l_{V2}}{\mathop {\textrm{sg}}} \left( {{\hat{\varepsilon }} _V^p} \right) - {l_{V3}}{\mathop {\textrm{sg}}} \left( {{\hat{\varepsilon }} _V^q} \right) , \end{aligned}$$

where ${l_{V1}},{l_{V2}},{l_{V3}}$ are the positve parameters to be designed.

### Altitude backstepping controller design

*Step 1:* Define the tracking error of altitude as

23 $$\begin{aligned} {e_h} = h - {h_{ref}}, \end{aligned}$$

Taking the time derivative of $e_h$ and substituting it into (6), one has

24 $$\begin{aligned} \dot{e}_h= V\gamma + {d_h} - \dot{h}_{ref} , . \end{aligned}$$

Define ${\varepsilon _h}$ as the absolute upper bound of ${d_h} - {\dot{h}_{ref}}$, the value of which is unknown. In order to enhance the robustness of the system, ${{\hat{\varepsilon }} _h}$ is used to estimate the value of ${\varepsilon _h}$ with the estimation error being ${{\tilde{\varepsilon }} _h} = {\varepsilon _h} - {{\hat{\varepsilon }} _h}$. The altitude virtual controller is designed as

25 $$\begin{aligned} {\gamma _d} = \frac{1}{V}\left[ { - {k_{h1}}{e_h} - {k_{h2}}{\mathop {\textrm{sg}}\nolimits } \left( {e_h^p} \right) - {k_{h3}}{\hbar _h} - {{{\hat{\varepsilon }} }_h}\tanh \left( {\frac{{{e_h}}}{{{\sigma _h}}}} \right) } \right] , \end{aligned}$$

where $k_{h1}, k_{h2}, k_{h3}, {\sigma _h}$ are the positve parameters to be designed. The switching function ${\hbar _h}$ is designed as

26 $$\begin{aligned} {\hbar _h} = \left\{ {\begin{array}{*{20}{l}} {{\mathop {\textrm{sg}}\nolimits } \left( {e_h^q} \right) ,}&{}{{\textrm{if}} |{{e_h}} |> {\tau _h},}\\ {\frac{{3 - q}}{2}\tau _h^{q - 1}{e_h} + \frac{{q - 1}}{2}\tau _h^{q - 3}e_h^3,}&{}{\textrm{others}}, \end{array}} \right. \end{aligned}$$

where ${\tau _h} > 0$ are the parameters to be designed.

#### Remark 2

By using the switching function (26), the singular value problem of infinite derivative when $e_h$ equal to zero can be avoided, and the switching point can be ensured to be smooth. Specifically, it can be written as follows

27 $$\begin{aligned} \mathop {\lim }\limits _{{e_h} \rightarrow - \tau _h^ - } {\hbar _h} =&\mathop {\lim }\limits _{{e_h} \rightarrow - \tau _h^ + } {\hbar _h} = - \tau _h^q, \mathop {\lim }\limits _{{e_h} \rightarrow \tau _h^ - } {\hbar _h} = \mathop {\lim }\limits _{{e_h} \rightarrow \tau _h^ + } {\hbar _h} = \tau _h^q,\nonumber \\ \mathop {\lim }\limits _{{e_h} \rightarrow - \tau _h^ - } {{\dot{\hbar }} _h} =&\mathop {\lim }\limits _{{e_h} \rightarrow - \tau _h^ + } {{\dot{\hbar }} _h} = q\tau _h^{q - 1}, \mathop {\lim }\limits _{{e_h} \rightarrow \tau _h^ - } {{\dot{\hbar }} _h} = \mathop {\lim }\limits _{{e_h} \rightarrow \tau _h^ + } {{\dot{\hbar }} _h} = q\tau _h^{q - 1},\nonumber \\ \mathop {\lim }\limits _{{e_h} \rightarrow {0^ - }} {{\dot{\hbar }} _h} =&\mathop {\lim }\limits _{{e_h} \rightarrow {0^ + }} {{\dot{\hbar }} _h} = q\tau _h^{q - 1}. \end{aligned}$$

Design the adaptive law as

28 $$\begin{aligned} {\dot{{\hat{\varepsilon }}} _h} = {e_h}\tanh \left( {\frac{{{e_h}}}{{{\sigma _h}}}} \right) - {l_{h1}}{{\hat{\varepsilon }} _h} - {l_{h2}}{\mathop {\textrm{sg}}} \left( {{\hat{\varepsilon }} _h^p}\right) - {l_{h3}}{\mathop {\textrm{sg}}} \left( {{\hat{\varepsilon }} _h^q}\right) , \end{aligned}$$

where ${l_{h1}},{l_{h2}},{l_{h3}}$ are the positve parameters to be designed.

*Step 2:* Define the tracking error of flight path angle as

29 $$\begin{aligned} {e_\gamma } = \gamma - {\gamma _d},. \end{aligned}$$

Combined with (6) and (29), the derivative of $e_\gamma$ is

30 $$\begin{aligned} {\dot{e}_\gamma } = {F_\gamma } + \alpha + {d_\gamma } - {{\dot{\gamma }} _d}. \end{aligned}$$

Define ${\varepsilon _\gamma }$ as the absolute upper bound of ${F_\gamma } + {d_\gamma } - {{\dot{\gamma }} _d}$, the value of which is unknown. In order to enhance the robustness of the system, ${{\hat{\varepsilon }} _\gamma }$ is used to estimate the value of ${\varepsilon _\gamma }$ with the estimation error being ${{\tilde{\varepsilon }} _\gamma } = {\varepsilon _\gamma } - {{\hat{\varepsilon }} _\gamma }$. The flight path angle virtual controller is designed as

31 $$\begin{aligned} {\alpha _d} = - {k_{\gamma 1}}{e_\gamma } - {k_{\gamma 2}}{\mathop {\textrm{sg}}\nolimits } \left( {e_\gamma ^p} \right) - {k_{\gamma 3}}{\hbar _\gamma } - {{\hat{\varepsilon }} _\gamma }\tanh \left( {\frac{{{z_\gamma }}}{{{\sigma _\gamma }}}} \right) , \end{aligned}$$

where $k_{\gamma 1}, k_{\gamma 2}, k_{\gamma 3}, {\sigma _\gamma }$ are the positve parameters to be designed. The switching function ${\hbar _\gamma }$ is designed as

32 $$\begin{aligned} {\hbar _\gamma } = \left\{ {\begin{array}{*{20}{l}} {{\mathop {\textrm{sg}}\nolimits } \left( {e_\gamma ^q} \right) ,}&{}{{\textrm{if}} |{{e_\gamma }} |> {\tau _\gamma },}\\ {\frac{{3 - q}}{2}\tau _\gamma ^{q - 1}{e_\gamma } + \frac{{q - 1}}{2}\tau _\gamma ^{q - 3}e_\gamma ^3,}&{}{\textrm{others}}, \end{array}} \right. \end{aligned}$$

where ${\tau _\gamma } > 0$ are the parameters to be designed.

To make angle of attack satisfy the preset constraint, let $\alpha _d$ pass the following saturation function

33 $$\begin{aligned} {\alpha _{ds}} = \left\{ {\begin{array}{*{20}{l}} {{\alpha _{dM}},}&{}\text {if}\ {{\alpha _d} > {\alpha _{dM}}},\\ {{\alpha _d},}&{}\text {if}\ {{\alpha _{dm}} \le {\alpha _d} \le {\alpha _{dM}}},\\ {{\alpha _{dm}},}&{}\text {if}\ {{\alpha _d} < {\alpha _{dm}}}, \end{array}} \right. \end{aligned}$$

where ${\alpha _{dM}}$ and ${\alpha _{dm}}$ are the user-designed upper and lower bounds, respcetively. To facilitate the derivation of stability analysis, the definition ${\bar{\alpha }} = \max \left\{ {|{{\alpha _{dm}}} |, |{{\alpha _{dM}}} |} \right\}$ is given.

Design the adaptive law as

34 $$\begin{aligned} {\dot{{\hat{\varepsilon }}} _\gamma } = {e_\gamma }\tanh \left( {\frac{{{e_\gamma }}}{{{\sigma _\gamma }}}} \right) - {l_{\gamma 1}}{{\hat{\varepsilon }} _\gamma } - {l_{\gamma 2}}{\mathop {\textrm{sg}}} \left( {{\hat{\varepsilon }} _\gamma ^p}\right) - {l_{\gamma 3}}{\mathop {\textrm{sg}}} \left( {{\hat{\varepsilon }} _\gamma ^q}\right) , \end{aligned}$$

where ${l_{\gamma 1}},{l_{\gamma 2}},{l_{\gamma 3}}$ are the positve parameters to be designed.

*Step 3:* Define the tracking error of angle of attack as

35 $$\begin{aligned} e_\alpha = \alpha - { \alpha _{ds}}. \end{aligned}$$

Combined with (6) and (35), the derivative of $e_\alpha$ is

36 $$\begin{aligned} {\dot{e}_\alpha } = {F_\alpha } + Q + {d_\alpha } - {{\dot{\alpha }} _{ds}}. \end{aligned}$$

Define ${\varepsilon _\alpha }$ as the absolute upper bound of ${F_\alpha } + {d_\alpha } - {{\dot{\alpha }} _{ds}} + {\Theta _\alpha }$, ${\Theta _\alpha }$ will be defined later. In order to enhance the robustness of the system, ${{\hat{\varepsilon }} _\alpha }$ is used to estimate the value of ${\varepsilon _\alpha }$ with the estimation error being ${{\tilde{\varepsilon }} _\alpha } = {\varepsilon _\alpha } - {{\hat{\varepsilon }} _\alpha }$. The angle of attack virtual controller is designed as

37 $$\begin{aligned} Q_{d} & = - k_{{\alpha 1}} \frac{{k^{2} \left( t \right) - \alpha ^{2} }}{{k^{2} \left( t \right)}}e_{\alpha } - \frac{{k_{{\alpha 2}} {\text{sg}}\left( {e_{\alpha }^{p} } \right)}}{{\left( {k^{2} \left( t \right) - \alpha ^{2} } \right)^{{\frac{{p - 1}}{2}}} }} - \frac{{k_{{\alpha 3}} \hbar _{\alpha } }}{{\left( {k^{2} \left( t \right) - \alpha ^{2} } \right)^{{\frac{{q - 1}}{2}}} }} \\ & \quad - \bar{\lambda }_{\alpha } e_{\alpha } - \hat{\varepsilon }_{\alpha } \tanh \left( {\frac{{e_{\alpha } }}{{\sigma _{\alpha } }}} \right) \\ \end{aligned}$$

where $k_{\alpha 1}, k_{\alpha 2}, k_{\alpha 3}, {\sigma _\alpha }$ are the positve parameters to be designed. The switching function ${\hbar _\alpha }$ is designed as

38 $$\begin{aligned} {\hbar _\alpha } = \left\{ {\begin{array}{*{20}{l}} {{\mathop {\textrm{sg}}\nolimits } \left( {e_\alpha ^q} \right) ,}&{}{{\textrm{if}} |{{e_\alpha }} |> {\tau _\alpha },}\\ {\frac{{3 - q}}{2}\tau _\alpha ^{q - 1}{e_\alpha } + \frac{{q - 1}}{2}\tau _\alpha ^{q - 3}e_\alpha ^3,}&{}{\textrm{others}}, \end{array}} \right. \end{aligned}$$

where ${\tau _\alpha } > 0$ are the parameters to be designed.

The time-varying gain function is designed as $\bar{\lambda }_{\alpha }$

39 $$\bar{\lambda }_{\alpha } = \sqrt {\left( {\frac{{\dot{k}_{a} \left( t \right)}}{{k_{a} \left( t \right)}}} \right)^{2} + \left( {\frac{{\dot{k}_{b} \left( t \right)}}{{k_{b} \left( t \right)}}} \right)^{2} + o_{\alpha } }$$

where ${o_\alpha }>0$ is the parameter to be designed.

The angle of attack constraint is an asymmetric time-varying function, which is expressed as

40 $$\begin{aligned} k\left( t \right) = \left\{ {\begin{array}{*{20}{l}} {{k_a}\left( t \right) ,}&{}\text {if}\ { {e_\alpha } < 0,}\\ {{k_b}\left( t \right) ,}&{}\text {others,} \end{array}} \right. \end{aligned}$$

where ${k_a}\left( t \right)$ and ${k_b}\left( t \right)$ is determined by aerodynamic coefficient analysis, the detailed process is shown in Sect. 2.2.

Design the adaptive law as

41 $$\begin{aligned} {{\dot{{\hat{\varepsilon }}} }_\alpha } = \frac{{e_\alpha {k^2}\left( t \right) }}{{ {{k^2}\left( t \right) - {\alpha ^2}} }} \tanh \left( {\frac{{{e_\alpha }}}{{{\sigma _\alpha }}}} \right) - {l_{\alpha 1}}{{{\hat{\varepsilon }} }_\alpha } - {l_{\alpha 2}}{\mathop {\textrm{sg}}} \left( {{\hat{\varepsilon }} _\alpha ^p} \right) - {l_{\alpha 3}}{\mathop {\textrm{sg}}} \left( {{\hat{\varepsilon }} _\alpha ^q} \right) , \end{aligned}$$

where ${l_{\alpha 1}},{l_{\alpha 2}},{l_{\alpha 3}}$ are the positve parameters to be designed.

*Step 4:* Define the tracking error of pitch rate as

42 $$\begin{aligned} e_Q = Q - { Q _{d}}. \end{aligned}$$

Combined with (6) and (42), the derivative of $e_Q$ is

43 $$\begin{aligned} {\dot{e}_Q} = {F_Q} + {g_Q}{\delta _{\textrm{e}}} + {d_Q} - {\dot{Q}_d}. \end{aligned}$$

Define ${\varepsilon _Q}$ as the absolute upper bound of ${F_Q} + {d_Q} - {\dot{Q}_d}$, the value of which is unknown. In order to enhance the robustness of the system, ${{\hat{\varepsilon }} _Q}$ is used to estimate the value of ${\varepsilon _Q}$ with the estimation error being ${{\tilde{\varepsilon }} _Q} = {\varepsilon _Q} - {{\hat{\varepsilon }} _Q}$. The actual controller is designed as

44 $$\begin{aligned} {\delta _{\textrm{e}}} = \frac{1}{{{g_Q}}}\left[ { - {k_{Q1}}{e_Q} - {k_{Q2}}{\mathop {\textrm{sg}}\nolimits } \left( {e_Q^p} \right) - {k_{Q3}}{\hbar _Q} - {{{\hat{\varepsilon }} }_Q}\tanh \left( {\frac{{{e_Q}}}{{{\sigma _Q}}}} \right) } \right] , \end{aligned}$$

where $k_{Q1}, k_{Q2}, k_{Q3}, {\sigma _Q}$ are the positve parameters to be designed. The switching function ${\hbar _Q}$ is designed as

45 $$\begin{aligned} {\hbar _Q} = \left\{ {\begin{array}{*{20}{l}} {{\mathop {\textrm{sg}}\nolimits } \left( {e_Q^q} \right) ,}&{}{{\textrm{if}} |{{e_Q}} |> {\tau _Q},}\\ {\frac{{3 - q}}{2}\tau _Q^{q - 1}{e_Q} + \frac{{q - 1}}{2}\tau _Q^{q - 3}e_Q^3,}&{}{\textrm{others}}, \end{array}} \right. \end{aligned}$$

where ${\tau _Q} > 0$ are the parameters to be designed.

Design the adaptive law as

46 $$\begin{aligned} {\dot{{\hat{\varepsilon }}} _Q} = {e_Q}\tanh \left( {\frac{{{e_Q}}}{{{\sigma _Q}}}} \right) - {l_{Q1}}{{\hat{\varepsilon }} _Q} - {l_{Q2}}{\mathop {\textrm{sg}}} \left( {{\hat{\varepsilon }} _Q^p }\right) - {l_{Q3}}{\mathop {\textrm{sg}}} \left( {{\hat{\varepsilon }} _Q^q}\right) , \end{aligned}$$

where ${l_{Q1}},{l_{Q2}},{l_{Q3}}$ are the positve parameters to be designed.

**Figure 10:**
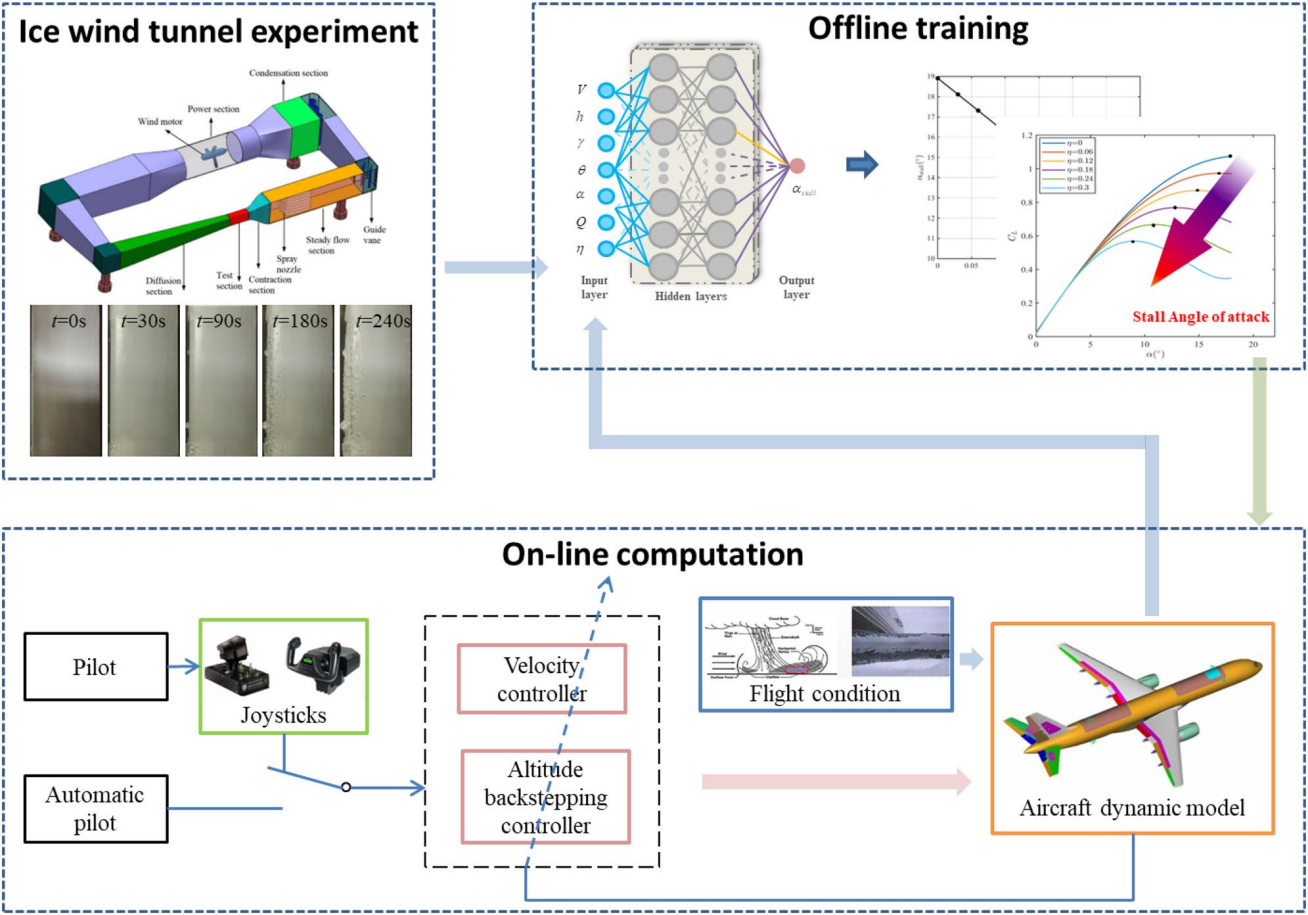
The block diagram of the proposed method.

The block diagram of the proposed method is shown as Fig. 10.

#### Remark 3

The disadvantages of the proposed method mainly include two aspects: First, although the neural network has a good fitting effect and can train the icing factor model accurately, it is not physically interpretable; Second, this method relies more on wind tunnel test, although the obtained aerodynamic data is more accurate, its cost is high. A valuable research direction is to combine wind tunnel and numerical calculation method to obtain icing aircraft aerodynamic data.

## Stability analysis

### Theorem 1

Consider the icing aircraft dynamics described by (1)–(4) with Assumptions 1–2, by the virtual control laws (25), (31), (37), by the actual control laws (20), (44), and by the parameter adaptation laws (22), (34), (41) and (46). For any $\zeta >$0, and bounded initial conditions satisfying $L\left( 0 \right) \le \zeta$, there exist design parameters $k_{i1}, k_{i2}, k_{i3}, {\sigma _i},{\tau _i},{l_{i1}},{l_{i2}},{l_{i3}}$ such that: i) all signals of the closed-loop system are semi-globally uniformly ultimately bounded; ii) the tracking errors and estimated errors converge to predefined compact set within fixed-time; iii) the angle of attack is kept within a preset asymmetric time-varying compact set determined by $k\left( t\right)$.

### Proof

The proof details are given in Online Appendix. $\square$

## Simulation results

### Simulation setup

This section verifies the effectiveness of the designed controller through simulation. The simulation step is set as $\Delta t = 0.005$s. The controller parameters are set as follows: $p = 1.{\textrm{1}},q = 0.{\textrm{9}},{k_{V1}} = 0.002,{k_{V2}} ={k_{V3}} = 0.0002, {k_{h1}} = 20,{k_{h2}} ={k_{h3}} = 2, {k_{\gamma 1}} = 0.15, {k_{\gamma 2}} = {k_{\gamma 3}} = 0.015, {k_{\alpha 1}} = 10, {k_{\alpha 2}} = {k_{\alpha 3}} = 1,{k_{Q 1}} = 25, {k_{Q 2}} = {k_{Q 3}} = 1, {\sigma _V} = {\sigma _h} = {\sigma _\gamma } = {\sigma _\alpha } = {\sigma _Q} = 40,{\tau _V} = {\tau _h} = {\tau _\gamma } = {\tau _\alpha } = {\tau _Q} = {\textrm{1}}{{\textrm{0}}^{ - 6}}, {l_{V1}} = {l_{h1}} = {l_{\gamma 1}} = {l_{\alpha 1}} = {l_{Q1}} = 0.1, {l_{V2}} = {l_{h2}} = {l_{\gamma 2}} = {l_{\alpha 2}} = {l_{Q2}} ={l_{V3}} = {l_{h3}} = {l_{\gamma 3}} = {l_{\alpha 3}} = {l_{Q3}} = 0.01$. The asymmetric time-varying angle of attack constraint is given by the deep neural network in real time. In practice, the inputs of flight controller are subject to saturation constraints, which are considered as $\Phi \in \left[ {0.05,1} \right] , {\delta _e} \in \left[ { - {{20}^ \circ },{{20}^ \circ }} \right]$. The initial state of the aircraft is shown in Table 1.

**Table 1:** The initial states of aircraft.

States	Values
Velocity *V*	150 m/s
Altitude *h*	400 m
Flight path angle $\gamma$	0
Pitch angle $\theta$	5$^ \circ$
Angle of attack $\alpha$	5 $^ \circ$
Pitch rate *Q*	0

The reference velocity and altitude signals are denoted by the following equations.

47 $$\begin{aligned} \left\{ \begin{array}{ll} V_{_{{ref}}}\left( t\right) &{} = V\left( 0\right) +\delta V_{\textrm{r}}\left( t\right) \\ h_{_{{ref}}}\left( t\right) &{} = h\left( 0\right) +\delta h_{\textrm{r}}\left( t\right) \end{array} \right. \end{aligned}$$

where $V\left( 0\right)$ and $h\left( 0\right)$ are the initial of velocity and altitude, respectively, and $\delta V_{\textrm{r}}\left( t\right)$ and $\delta h_{\textrm{r}}\left( t\right)$ are the time-varying components of the reference signals. The time-varying components $\delta V_{\textrm{r}}\left( t\right)$ and $\delta h_{\textrm{r}}\left( t\right)$ are generated by passing command signals $\delta V_{\textrm{c}}\left( t\right)$ and $\delta h_{\textrm{c}}\left( t\right)$ through a 2nd-order filter, respectively. Hence, one has

48 $$\begin{aligned} \left\{ \begin{array}{ll} \delta V_{\textrm{r}}\left( t\right) &{} = \mathcal {L}^{-1}\left[ \frac{{0.0.0009}}{{{s^2} + 0.057s + 0.0.0009}}\right] * \delta V_{\textrm{c}} \\ \delta h_{\textrm{r}}\left( t\right) &{} = \mathcal {L}^{-1}\left[ \frac{{0.0.0009}}{{{s^2} + 0.057s + 0.0.0009}}\right] * \delta h_{\textrm{c}} \end{array} \right. \end{aligned}$$

where $\mathcal {L}^{-1}\left[ \cdot \right]$ denotes the inverse of Laplacian transform, “*” is the convolution operation, $\delta V_{\textrm{c}}$ is the commanded velocity, and $\delta h_{\textrm{c}}$ is the commanded altitude.

**Figure 11:**
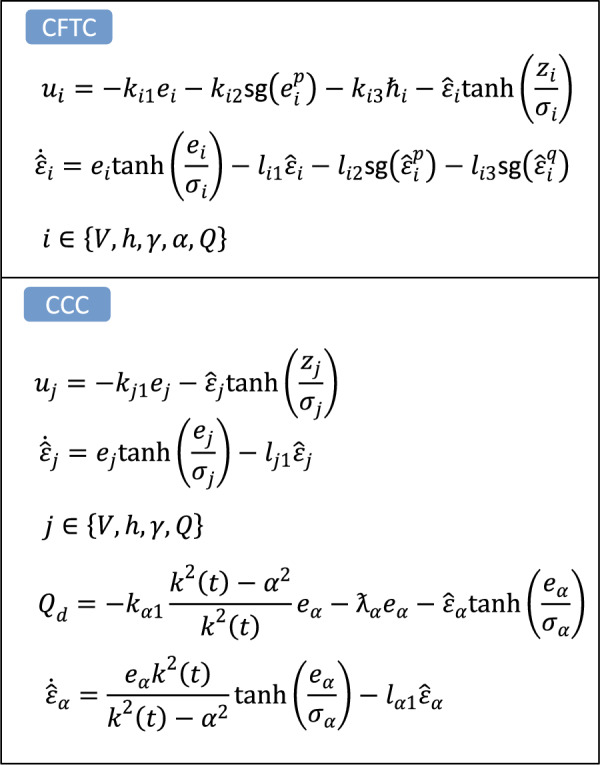
The structural frameworks of CCC and CFTC.

In order to demonstrate the superiority of the proposed method, the simulation results of the proposed method are compared with those of the conventional constrained control (CCC)^20^ and those of the conventional fixed-time control (CFTC)^31^. The structural frameworks of CCC and CFTC is shown as Fig. 11.

### Scenario 1: Dynamic icing process

In this scenario, the airfoil freezes from *t*=0s and reaches the most severe freezing state $\eta$=0.3 at *t*=240s. When *t* is between 50s and 60s, the pitch angle is disturbed by ${\Delta _\theta }{\mathrm{= 6}}\left[ {1 - {\textrm{exp}}\left( { - \frac{1}{5}t} \right) } \right]$deg.

**Figure 12:**
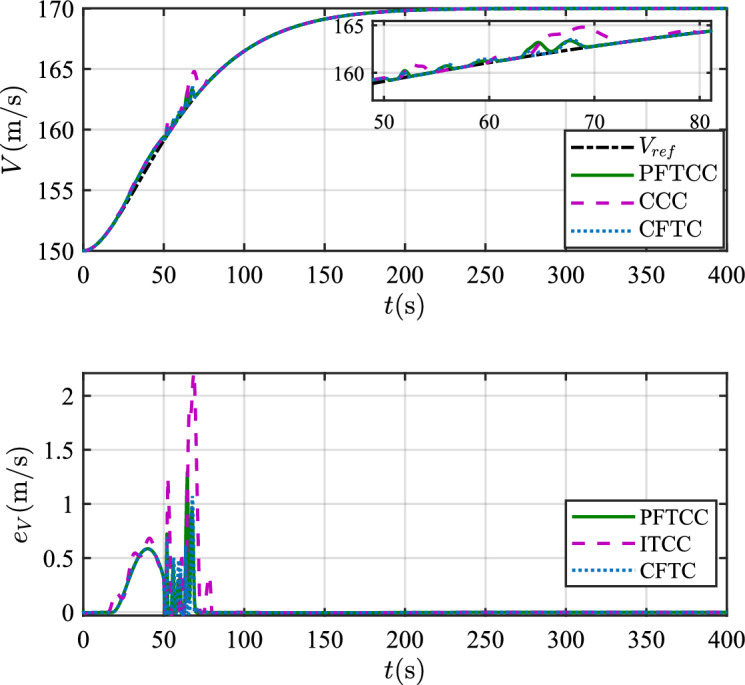
The velocity tracking performance.

**Figure 13:**
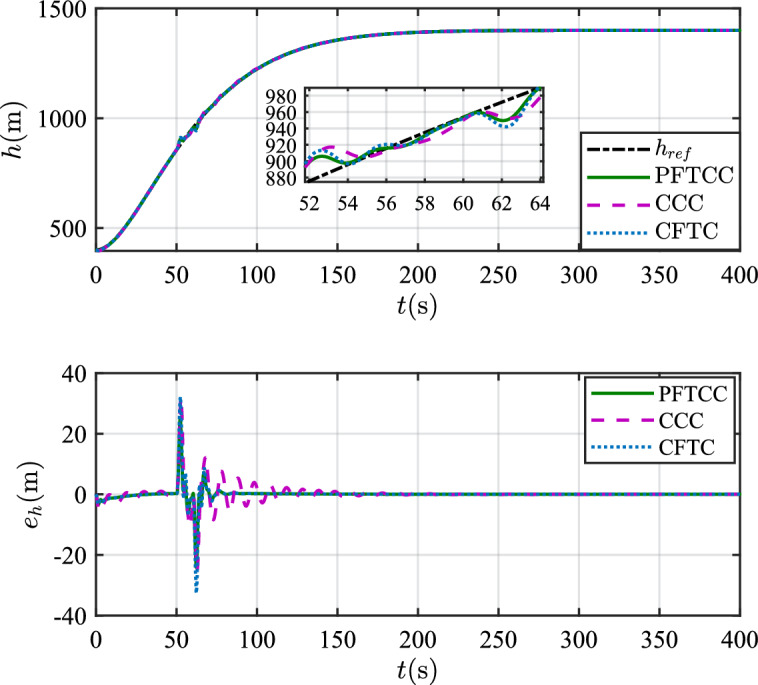
The altitude tracking performance.

**Figure 14:**
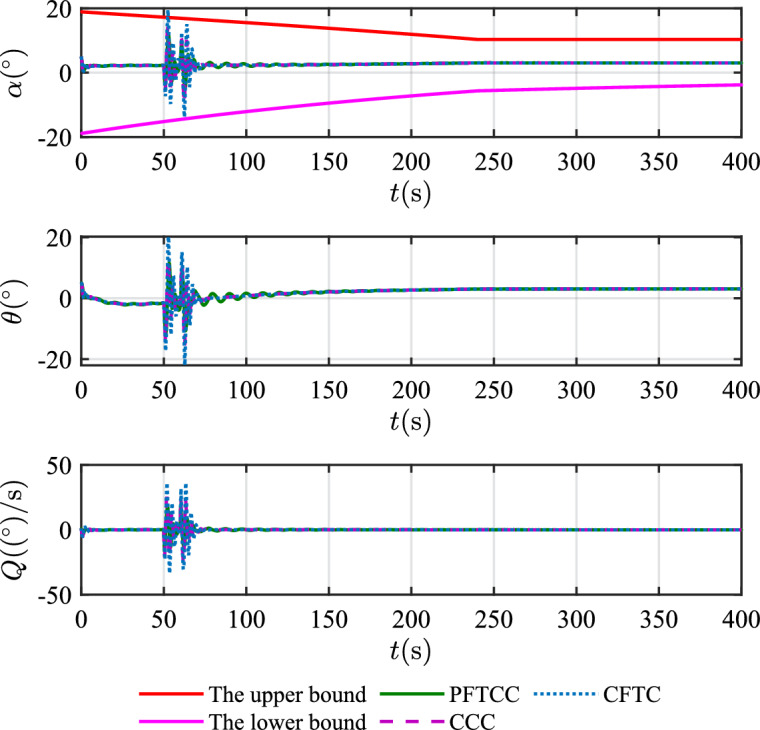
The attitude angles.

**Figure 15:**
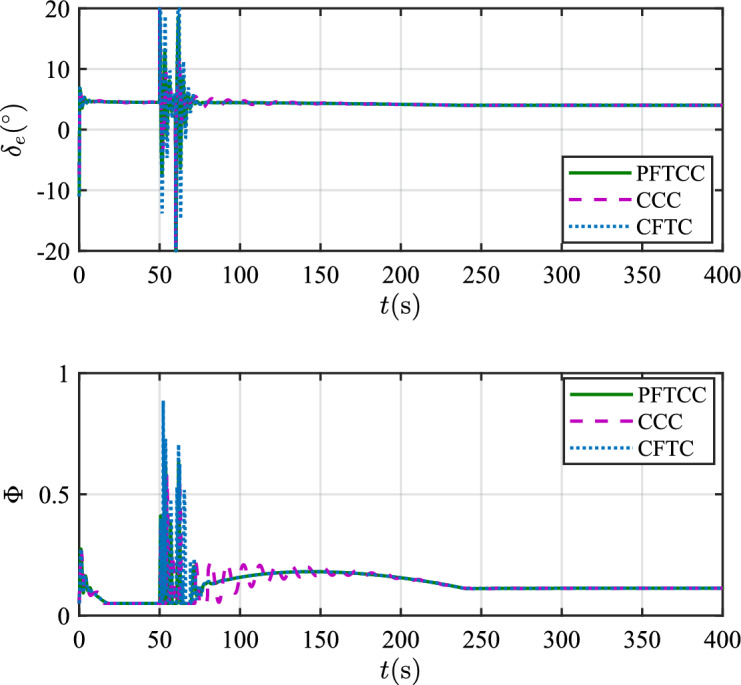
The control inputs.

**Figure 16:**
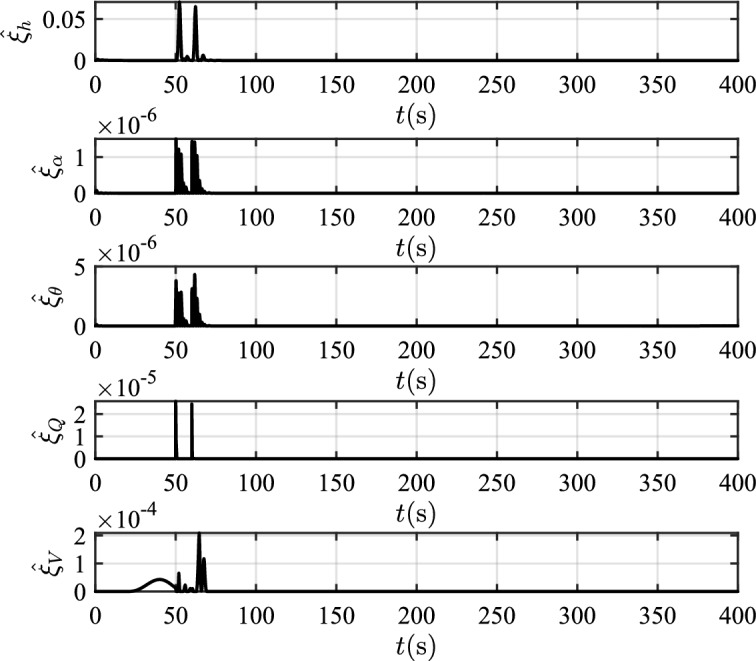
The values of adaptive parameters.

**Table 2:** The squares sum of tracking errors for scenario 1.

Performance indicators	CCC	CFTC	PFTCC
$\int {e_V^2} {\textrm{d}}t$	6231.50	1889.86	1582.13
$\int {e_h^2} {\textrm{d}}t$	846222.87	625411.82	373919.99

**Table 3:** The squares sum of tracking errors for scenario 2.

Performance indicators	CCC	CFTC	PFTCC
$\int {e_V^2} {\textrm{d}}t$	6233.12	1890.86	1583.16
$\int {e_h^2} {\textrm{d}}t$	846230.01	625412.12	373920.30

In scenario 1, the velocity and tracking error of velocity are shown in Fig. 12, and the altitude and tracking error of altitude are shown in Fig. 13. It can be seen that in the case of dynamic icing process, the overshoot of tracking error of velocity and tracking error of altitude produced by the proposed method are smaller than the overshoot produced by the other two methods. Figure 14 shows the flight path angle, angle of attack and pitch rate. The angle of attack can be kept within the preset constraint by using the PFTCC and CCC, while the constraint is violated via the CFTC. The oscillation of attitude angles via the proposed method are smaller. The fuel equivalent ratio and elevator deflection are shown in Fig. 15, indicating that the control input with the proposed method are quickly stabilized. It can be seen from Fig. 16 that the adaptive parameters of the robustness terms are bounded. In order to compare the control performance of the three methods more obviously, the squares sum of velocity tracking errors and squares sum of altitude tracking errors of the three methods are listed in Table 2. It can be seen that the squares sum of velocity tracking errors and squares sum of altitude tracking errors obtained by the proposed method are smaller than those obtained by the other two methods. Therefore, the proposed method has better control performance.

### Scenario 2: deicing process

In this scenario, the icing process has completed. When *t*=240s, the deicing device is turned on and all the ice is completely removed after 160s. It is assumed that the icing intensity changes linearly during the deicing process. When *t* is between 50s and 60s, the pitch angle is disturbed by ${\Delta _\theta }{\mathrm{= 6}}\left[ {1 - {\textrm{exp}}\left( { - \frac{1}{5}t} \right) } \right]$deg.

**Figure 17:**
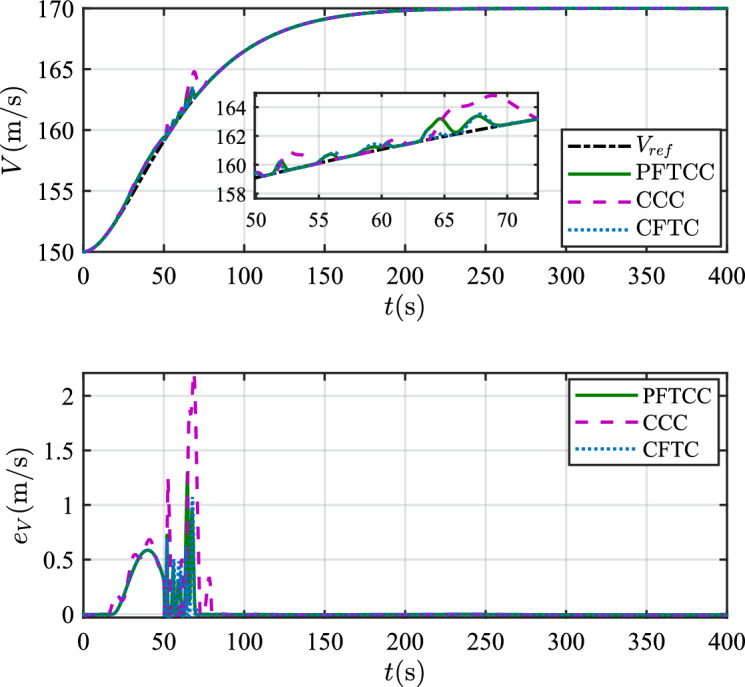
The velocity tracking performance.

**Figure 18:**
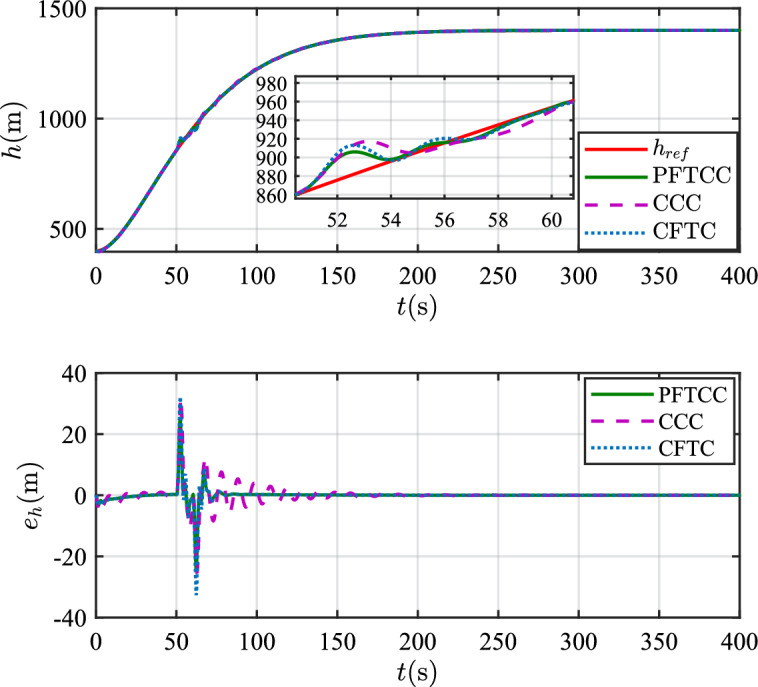
The altitude tracking performance.

**Figure 19:**
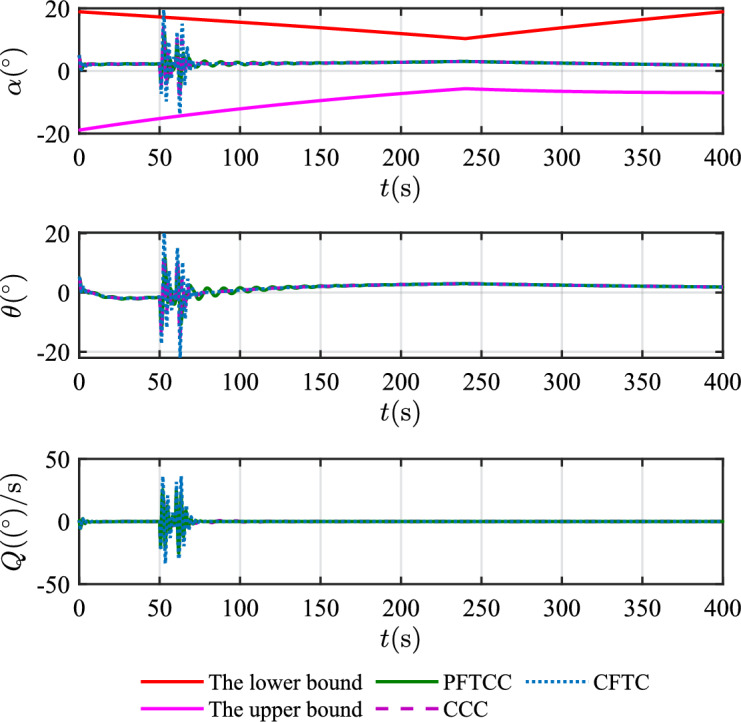
The attitude angles.

**Figure 20:**
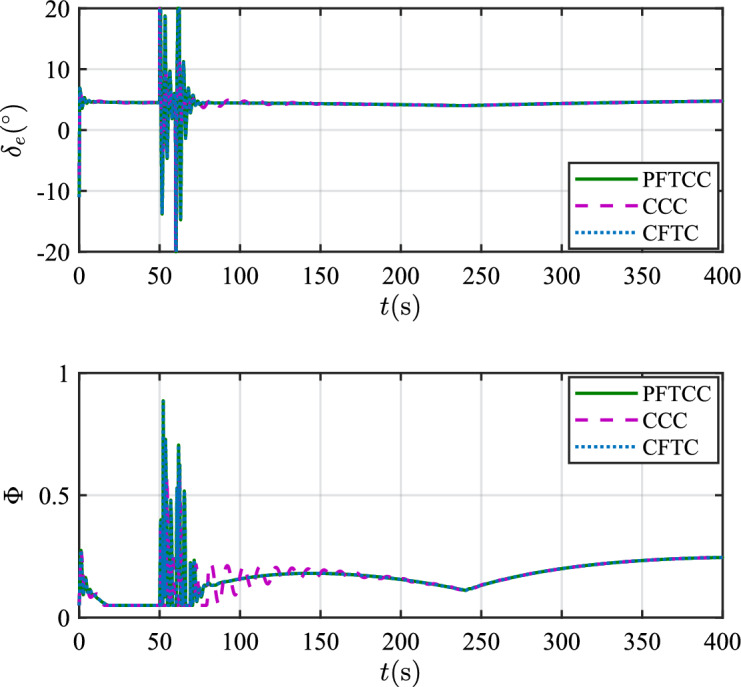
The control inputs.

**Figure 21:**
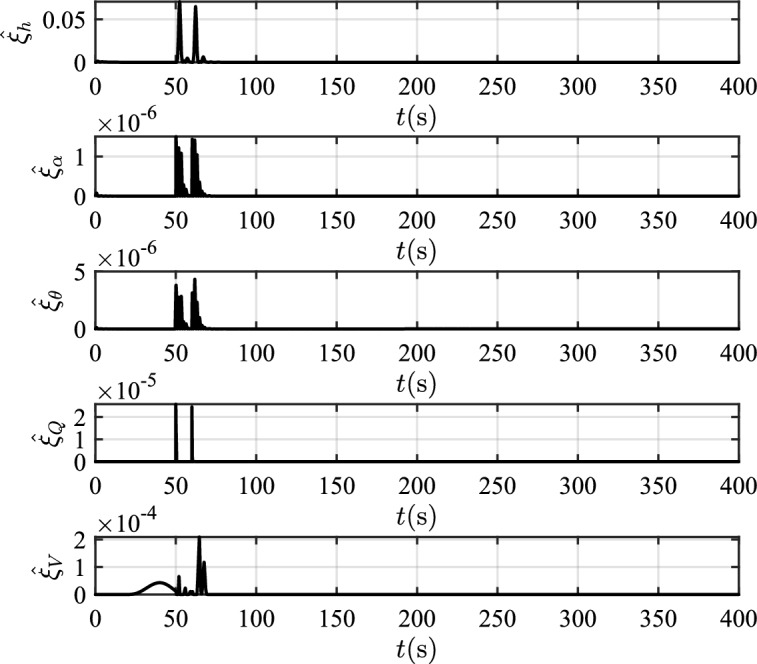
The values of adaptive parameters.

In scenario 2, the velocity and tracking error of velocity are shown in Fig. 17, and the altitude and tracking error of altitude are shown in Fig. 18. It can be seen that the proposed method can not only make the velocity and altitude track their own reference commands faster, but also make the fluctuation of the tracking errors smaller when affected by the external disturbance. The flight path angle, angle of attack and pitch rate are shown in Fig. 19. After the deicing device is started, the stall angle of attack increases because the icing intensity decreases gradually. The angle of attack can be kept within the preset constraint by using the PFTCC and CCC, while the constraint is violated via the CFTC. Figure 20 shows the fuel equivalent ratio and elevator deflection. In Fig. 21, the boundedness of the adaptive parameters of the proposed method is demonstrated. The squares sum of velocity tracking errors and squares sum of altitude tracking errors in scenario 2 are shown in Table 3, indicating that the proposed method still has better control performance in the presence of external disturbance.

### Ethical approval

This research does not include any human participants and/or Animals

## Conclusion

This paper presents a fixed-time robust control method for aircraft with angle of attack constraint considering dynamic icing process, asymmetric time-varying constraint of angle of attack and external disturbance. Through theoretical analysis and simulation verification, the main conclusions are as follows:

- A fixed-time non-singular robust tracking controller is designed to ensure that the tracking errors and estimation errors converge within fixed-time, so that the singularity problem inherent in the conventional fixed-time control is avoided.
- The asymmetric time-varying constraint of angle of attack is assured not to be violated in a direct way by utilizing the integral-type barrier function.
- The variation rule between the flight states, icing intensity and stall angle of attack is obtained by applying deep neural network.
- The proposed controller has better control performance compared with the conventional fixed-time control method and the conventional angle of attack-constrained control.

### Supplementary Information

Supplementary Information.

## Data Availability

The datasets generated during and/or analysed during the current study are available from the corresponding author on reasonable request.
