# Supplementary material for: Fixed-time angle of attack constrained control for aircraft considering dynamic icing process
Source: Sci Rep. 2024 Mar 4;14:1388. doi: 10.1038/s41598-023-50038-y (PMC11251185; doi:10.1038/s41598-023-50038-y)
Supplement: Supplementary file 1 — Supplementary Information. [file 41598_2023_50038_MOESM1_ESM.pdf]

Appendix

Proof of Theorem 1:

Construct the Lyapunov function candidate as

$$L = L_V + L_h + L_\gamma + L_\alpha + L_Q, \tag{1}$$

where

$$L_i = \frac{1}{2}e_i^2 + \frac{1}{2}\tilde{\varepsilon}_i^2, i \in \{V, h, \gamma, Q\}, \tag{2}$$

$$L_\alpha = \int_0^{e_\alpha} \frac{\varpi k^2(t)}{k^2(t) - (\varpi + \alpha_{ds})^2} d\varpi + \frac{1}{2}\tilde{\varepsilon}_\alpha^2, \tag{3}$$

where the substitution $\varpi = we_\alpha$ with $w > 0$ being the parameter to be designed.

Due to that $\alpha_{ds} \leq \bar{\alpha}$, one has

$$\frac{1}{2}e_\alpha^2 \leq L_\alpha \leq e_\alpha^2 \int_0^1 \frac{wk^2(t)}{k^2(t) - (we_\alpha + \operatorname{sign}(e_\alpha)\bar{\alpha})^2} dw. \tag{4}$$

For the sake of analysis, the following function is defined

$$\psi(t) = \frac{\varpi k^2(t)}{k^2(t) - (\varpi + \alpha_{ds})^2}. \tag{5}$$

The time derivative of L_α is derived as

$$\begin{aligned}\dot{L}_\alpha &= \lim_{\Delta t \to 0} \frac{L_\alpha(t + \Delta t) - L_\alpha(t)}{\Delta t} - \tilde{\varepsilon}_\alpha \dot{\hat{\varepsilon}}_\alpha \\ &= \lim_{\Delta t \to 0} \frac{1}{\Delta t} \int_{e_\alpha(t)}^{e_\alpha(t+\Delta t)} \psi(t + \Delta t) d\varpi \\ &\quad + \lim_{\Delta t \to 0} \frac{1}{\Delta t} \int_0^{e_\alpha(t)} (\psi(t + \Delta t) - \psi(t)) d\varpi - \tilde{\varepsilon}_\alpha \dot{\hat{\varepsilon}}_\alpha,\end{aligned} \tag{6}$$

With the help of the integral mean value theorem, we obtain

$$\begin{aligned}\dot{L}_\alpha &= \lim_{\Delta t \to 0} \psi(\kappa) \frac{e_\alpha(t + \Delta t) - e_\alpha(t)}{\Delta t} \\ &\quad + \int_0^{e_\alpha(t)} \lim_{\Delta t \to 0} \frac{\psi(t + \Delta t) - \psi(t)}{\Delta t} d\varpi - \tilde{\varepsilon}_\alpha \dot{\hat{\varepsilon}}_\alpha \\ &= \dot{e}_\alpha(t)\psi(t) + \int_0^{e_\alpha(t)} \lim_{\Delta t \to 0} \frac{d\psi(t)}{dt} d\varpi - \tilde{\varepsilon}_\alpha \dot{\hat{\varepsilon}}_\alpha,\end{aligned} \tag{7}$$

where $\kappa \in (e_\alpha(t), e_\alpha(t + \Delta t))$.

Further, the time derivative of L_α satisfies

$$\begin{aligned}\dot{L}_\alpha &= \frac{e_\alpha k^2(t)}{k^2(t) - \alpha^2} \dot{e}_\alpha + \dot{\alpha}_{ds} \int_0^{e_\alpha(t)} \frac{\partial}{\partial \alpha_d} \frac{\varpi k^2(t)}{k^2(t) - (\varpi + \alpha_d)^2} d\varpi \\ &\quad + \dot{k}(t) \int_0^{e_\alpha(t)} \frac{\partial}{\partial k(t)} \frac{\varpi k^2(t)}{k^2(t) - (\varpi + \alpha_d)^2} d\varpi - \tilde{\varepsilon}_\alpha \dot{\hat{\varepsilon}}_\alpha.\end{aligned} \tag{8}$$

The second term in (8) can be derived as

$$\begin{aligned}&\int_0^{e_\alpha(t)} \frac{\partial}{\partial \alpha_{ds}} \frac{\varpi k^2(t)}{k^2(t) - (\varpi + \alpha_{ds})^2} d\varpi \\ &= e_\alpha \left[\frac{k^2(t)}{k^2(t) - \alpha^2} - \Gamma(e_\alpha, \alpha_{ds}, k(t)) \right].\end{aligned} \tag{9}$$

The third term in (8) can be derived as

$$
\begin{aligned}
&\int_0^{e_\alpha(t)} \frac{\partial}{\partial k(t)} \frac{\varpi k^2(t)}{k^2(t)-(\varpi+\alpha_{ds})^2} d\varpi \\
&= \int_0^{e_\alpha(t)} -\varpi(\varpi+\alpha_{ds})\, d\frac{k(t)}{k^2(t)-(\varpi+\alpha_{ds})^2} \\
&= \frac{-e_\alpha(e_\alpha+\alpha_{ds})k(t)}{k^2(t)-(e_\alpha+\alpha_{ds})^2} + \int_0^{e_\alpha(t)} \frac{(2\varpi+\alpha_{ds})k(t)}{k^2(t)-(\varpi+\alpha_{ds})^2} d\varpi \\
&= e_\alpha\left[\frac{-(e_\alpha+\alpha_{ds})k(t)}{k^2(t)-(e_\alpha+\alpha_{ds})^2} + \int_0^1 \frac{(2we_\alpha+\alpha_{ds})k(t)}{k^2(t)-(we_\alpha+\alpha_{ds})^2} dw\right] \\
&= e_\alpha\left[\frac{-e_\alpha k(t)}{k^2(t)-(e_\alpha+\alpha_{ds})^2} + \Xi(e_\alpha,\alpha_{ds},k(t))\right].
\end{aligned} \tag{10}
$$

Then the time derivative of L_α can be rewritten as

$$
\begin{aligned}
\dot{L}_\alpha =& \frac{e_\alpha k^2(t)}{k^2(t)-\alpha^2}\dot{e}_\alpha + \dot{\alpha}_{ds}e_\alpha\left[\frac{k^2(t)}{k^2(t)-\alpha^2} - \Gamma(e_\alpha,\alpha_{ds},k(t))\right] \\
&+ \dot{k}(t)e_\alpha\left[\frac{-e_\alpha k(t)}{k^2(t)-(e_\alpha+\alpha_{ds})^2} + \Xi(e_\alpha,\alpha_{ds},k(t))\right] - \tilde{\varepsilon}_\alpha\dot{\hat{\varepsilon}}_\alpha \\
=& \frac{e_\alpha k^2(t)}{k^2(t)-\alpha^2}(F_\alpha+e_Q+Q_d+d_\alpha) - \dot{\alpha}_{ds}e_\alpha\Gamma(e_\alpha,\alpha_{ds},k(t)) \\
&- \frac{e_\alpha^2 k(t)\dot{k}(t)}{k^2(t)-(e_\alpha+\alpha_{ds})^2} + \dot{k}(t)e_\alpha\Xi(e_\alpha,\alpha_{ds},k(t)) - \tilde{\varepsilon}_\alpha\dot{\hat{\varepsilon}}_\alpha.
\end{aligned} \tag{11}
$$

Define

$$
\begin{aligned}
\Theta_\alpha =& -\frac{k^2(t)-\alpha^2}{k^2(t)}\dot{\alpha}_{ds}\Gamma(e_\alpha,\alpha_{ds},k(t)) \\
&+ \frac{k^2(t)-\alpha^2}{k^2(t)}\dot{k}(t)\,\Xi(e_\alpha,\alpha_{ds},k(t)),
\end{aligned} \tag{12}
$$

in view of ε_α, we have

$$
\dot{L}_\alpha \le \frac{\varepsilon_\alpha k^2(t)}{k^2(t)-\alpha^2}|e_\alpha| + \frac{e_\alpha k^2(t)}{k^2(t)-\alpha^2}\left[e_Q+Q_d+\frac{\dot{k}(t)}{k(t)}e_\alpha\right] - \tilde{\varepsilon}_\alpha\dot{\hat{\varepsilon}}_\alpha. \tag{13}
$$

Substituting control controller of AOA and adaptive law of AOA into (13) yields

$$
\begin{aligned}
\dot{L}_\alpha \leq & \frac{\varepsilon_\alpha k^2(t)}{k^2(t)-\alpha^2}|e_\alpha| + \frac{e_\alpha e_Q k^2(t)}{k^2(t)-\alpha^2} - \frac{\tilde{\varepsilon}_\alpha k^2(t)}{k^2(t)-\alpha^2} e_\alpha \tanh\left(\frac{e_\alpha}{\sigma_\alpha}\right) \\
& - \frac{\hat{\varepsilon}_\alpha k^2(t)}{k^2(t)-\alpha^2} e_\alpha \tanh\left(\frac{e_\alpha}{\sigma_\alpha}\right) + \frac{k^2(t)}{k^2(t)-\alpha^2}\left[-\left(\lambda_\alpha - \frac{\dot{k}(t)}{k(t)}\right) e_\alpha^2\right. \\
& \left. - k_{\alpha 1}\frac{k^2(t)-\alpha^2}{k^2(t)} e_\alpha^2 - \frac{k_{\alpha 2}\operatorname{sg}\left(e_\alpha^p\right) e_\alpha}{\left(k^2(t)-\alpha^2\right)^{\frac{p-1}{2}}} - \frac{k_{\alpha 3}\hbar_\alpha e_\alpha}{\left(k^2(t)-\alpha^2\right)^{\frac{q-1}{2}}}\right] \\
& + l_{\alpha 1}\tilde{\varepsilon}_\alpha \hat{\varepsilon}_\alpha + l_{\alpha 2}\tilde{\varepsilon}_\alpha \operatorname{sg}\left(\hat{\varepsilon}_\alpha^p\right) + l_{\alpha 3}\tilde{\varepsilon}_\alpha \operatorname{sg}\left(\hat{\varepsilon}_\alpha^q\right) \\
= & \frac{\varepsilon_\alpha k^2(t)}{k^2(t)-\alpha^2}\left[|e_\alpha| - e_\alpha \tanh\left(\frac{e_\alpha}{\sigma_\alpha}\right)\right] + \frac{e_\alpha e_Q k^2(t)}{k^2(t)-\alpha^2} \\
& + \frac{k^2(t)}{k^2(t)-\alpha^2}\left[-\left(\lambda_\alpha - \frac{\dot{k}(t)}{k(t)}\right) e_\alpha^2 - k_{\alpha 1}\frac{k^2(t)-\alpha^2}{k^2(t)} e_\alpha^2\right. \\
& \left. - \frac{k_{\alpha 2}\operatorname{sg}\left(e_\alpha^p\right) e_\alpha}{\left(k^2(t)-\alpha^2\right)^{\frac{p-1}{2}}} - \frac{k_{\alpha 3}\hbar_\alpha e_\alpha}{\left(k^2(t)-\alpha^2\right)^{\frac{q-1}{2}}}\right] + l_{\alpha 1}\tilde{\varepsilon}_\alpha \hat{\varepsilon}_\alpha \\
& + l_{\alpha 2}\tilde{\varepsilon}_\alpha \operatorname{sg}\left(\hat{\varepsilon}_\alpha^p\right) + l_{\alpha 3}\tilde{\varepsilon}_\alpha \operatorname{sg}\left(\hat{\varepsilon}_\alpha^q\right).
\end{aligned} \tag{14}
$$

According the definition of controller design, one has

$$
-\left(\lambda_\alpha - \frac{\dot{k}(t)}{k(t)}\right) \leq 0. \tag{15}
$$

Recalling Lemma 5, the derivative of L_α is further estimated to

$$
\begin{aligned}
\dot{L}_\alpha \leq & \frac{\kappa \varepsilon_\alpha \sigma_\alpha k^2(t)}{k^2(t)-\alpha^2} + \frac{e_\alpha e_Q k^2(t)}{k^2(t)-\alpha^2} + \frac{k^2(t)}{k^2(t)-\alpha^2}\left[-k_{\alpha 1}\frac{k^2(t)-\alpha^2}{k^2(t)} e_\alpha^2\right. \\
& \left. - \frac{k_{\alpha 2}\operatorname{sg}\left(e_\alpha^p\right) e_\alpha}{\left(k^2(t)-\alpha^2\right)^{\frac{p-1}{2}}} - \frac{k_{\alpha 3}\hbar_\alpha e_\alpha}{\left(k^2(t)-\alpha^2\right)^{\frac{q-1}{2}}}\right] + l_{\alpha 1}\tilde{\varepsilon}_\alpha \hat{\varepsilon}_\alpha \\
& + l_{\alpha 2}\tilde{\varepsilon}_\alpha \operatorname{sg}\left(\hat{\varepsilon}_\alpha^p\right) + l_{\alpha 3}\tilde{\varepsilon}_\alpha \operatorname{sg}\left(\hat{\varepsilon}_\alpha^q\right).
\end{aligned} \tag{16}
$$

Then the time derivative of L can be estimated by

$$
\begin{aligned}
\dot{L} = & \dot{L}_\alpha + \sum_{i \in \{V,h,\gamma,Q\}}\left[-k_{i1}e_i^2 - k_{i2}\left(e_i^2\right)^{\frac{p+1}{2}} - k_{i3}e_i\hbar_i + 2l_{i1}\hat{\varepsilon}_i\tilde{\varepsilon}_i\right. \\
& \left. + l_{i2}\operatorname{sg}\left(\hat{\varepsilon}_i^p\right)\tilde{\varepsilon}_i + l_{i3}\operatorname{sg}\left(\hat{\varepsilon}_i^q\right)\tilde{\varepsilon}_i\right] + \varepsilon_i\left(|e_i| - e_i\tanh\left(\frac{e_i}{\sigma_i}\right)\right)\Bigg] \\
& + e_h e_\gamma + e_\gamma e_\alpha \\
\leq & \dot{L}_\alpha + \sum_{i \in \{V,h,\gamma,Q\}}\left[-k_{i1}e_i^2 - k_{i2}\left(e_i^2\right)^{\frac{p+1}{2}} - k_{i3}e_i\hbar_i + 2l_{i1}\hat{\varepsilon}_i\tilde{\varepsilon}_i\right. \\
& \left. + l_{i2}\operatorname{sg}\left(\hat{\varepsilon}_i^p\right)\tilde{\varepsilon}_i + l_{i3}\operatorname{sg}\left(\hat{\varepsilon}_i^q\right)\tilde{\varepsilon}_i\right] + e_h e_\gamma + e_\gamma e_\alpha + \kappa \varepsilon_i \sigma_i.
\end{aligned} \tag{17}
$$

According to Young's inequality, one has

$$
e_h e_\gamma \leq \frac{e_h^2}{2} + \frac{e_\gamma^2}{2}, \tag{18}
$$

$$
e_\gamma e_\alpha \leq \frac{e_\gamma^2}{2} + \frac{e_\alpha^2}{2}, \tag{19}
$$

$$
\hat{\varepsilon}_i\tilde{\varepsilon}_i = \left(\varepsilon_i - \tilde{\varepsilon}_i\right)\tilde{\varepsilon}_i = \varepsilon_i\tilde{\varepsilon}_i - \tilde{\varepsilon}_i^2 \leq \frac{1}{2}\varepsilon_i^2 - \frac{1}{2}\tilde{\varepsilon}_i^2. \tag{20}
$$

It follows from Lemmas 2-3, one obtains

$$\begin{aligned}\mathrm{sg}\left(\hat{\varepsilon}_i^{p}\right)\tilde{\varepsilon}_i =&\mathrm{sg}\left(\hat{\varepsilon}_i^{p}\right)\left(\varepsilon_i-\hat{\varepsilon}_i\right)\leq\frac{1}{1+p}\left[\left(\varepsilon_i^2\right)^{\frac{p+1}{2}}-\left(\varepsilon_i-\tilde{\varepsilon}_i\right)^{p+1}\right]\\ \leq&\frac{1}{1+p}\left[2\left(\varepsilon_i^2\right)^{\frac{p+1}{2}}-\left(\tilde{\varepsilon}_i^2\right)^{\frac{p+1}{2}}\right],\end{aligned}\tag{21}$$

$$\begin{aligned}\mathrm{sg}\left(\hat{\varepsilon}_i^{q}\right)\tilde{\varepsilon}_i =&\mathrm{sg}\left(\hat{\varepsilon}_i^{q}\right)\left(\varepsilon_i-\hat{\varepsilon}_i\right)\leq\frac{1}{1+q}\left[\left(\varepsilon_i^2\right)^{\frac{q+1}{2}}-\left(\varepsilon_i-\tilde{\varepsilon}_i\right)^{q+1}\right]\\ \leq&\frac{1}{1+q}\left[2\left(\varepsilon_i^2\right)^{\frac{q+1}{2}}-\left(\tilde{\varepsilon}_i^2\right)^{\frac{q+1}{2}}\right],\end{aligned}\tag{22}$$

Substituting (21)-(22) into (17) arrives

$$\begin{aligned}\dot{L}\leq&\sum_{i\in\{V,h,\gamma,Q\}}\Bigg[-k_{i2}\left(e_i^2\right)^{\frac{p+1}{2}}-k_{i3}e_i\hbar_i-l_{i1}\tilde{\varepsilon}_i^2-\frac{l_{i2}}{1+p}\left(\tilde{\varepsilon}_i^2\right)^{\frac{p+1}{2}}\\ &-\frac{l_{i3}}{1+q}\left(\tilde{\varepsilon}_i^2\right)^{\frac{q+1}{2}}+\frac{2l_{i2}}{1+p}\left(\varepsilon_i^2\right)^{\frac{p+1}{2}}+\frac{2l_{i3}}{1+q}\left(\varepsilon_i^2\right)^{\frac{q+1}{2}}+l_{i1}\varepsilon_i^2\\ &+\kappa\sigma_i\varepsilon_i\Bigg]+\frac{k^2(t)}{k^2(t)-\alpha^2}\left[-k_{\alpha2}\left(e_\alpha^2\right)^{\frac{p+1}{2}}-k_{\alpha3}e_\alpha\hbar_\alpha\right]-l_{\alpha1}\tilde{\varepsilon}_\alpha^2\\ &-\frac{l_{\alpha2}}{1+p}\left(\tilde{\varepsilon}_\alpha^2\right)^{\frac{p+1}{2}}-\frac{l_{\alpha3}}{1+q}\left(\tilde{\varepsilon}_\alpha^2\right)^{\frac{q+1}{2}}+l_{\alpha1}\varepsilon_\alpha^2+\frac{2l_{\alpha2}}{1+p}\left(\varepsilon_\alpha^2\right)^{\frac{p+1}{2}}\\ &+\frac{2l_{\alpha3}}{1+q}\left(\varepsilon_\alpha^2\right)^{\frac{q+1}{2}}-k_{V1}e_V^2-\left(k_{h1}-\frac{1}{2}\right)e_h^2-\left(k_{\gamma1}-1\right)e_\gamma^2\\ &-\left(k_{\alpha1}-\frac{k^2(t)}{k^2(t)-\alpha^2}-\frac{1}{2}\right)e_\alpha^2-\left(k_{Q1}-\frac{k^2(t)}{2\left(k^2(t)-\alpha^2\right)}\right)e_Q^2.\end{aligned}\tag{23}$$

According to the design in controller design, the inequation (23) can be divided into the following two cases.
Case 1: When $|e_\bullet|>\tau_\bullet$, the inequation (23) can be written as

$$\begin{aligned}\dot{L}\leq&\sum_{i\in\{V,h,\gamma,Q\}}\Bigg[-k_{i2}\left(e_i^2\right)^{\frac{p+1}{2}}-k_{i3}\left(e_i^2\right)^{\frac{q+1}{2}}-l_{i1}\tilde{\varepsilon}_i^2-\frac{l_{i2}}{1+p}\left(\tilde{\varepsilon}_i^2\right)^{\frac{p+1}{2}}\\ &-\frac{l_{i3}}{1+q}\left(\tilde{\varepsilon}_i^2\right)^{\frac{q+1}{2}}+\frac{2l_{i2}}{1+p}\left(\varepsilon_i^2\right)^{\frac{p+1}{2}}+\frac{2l_{i3}}{1+q}\left(\varepsilon_i^2\right)^{\frac{q+1}{2}}+l_{i1}\varepsilon_i^2\\ &+\kappa\sigma_i\varepsilon_i\Bigg]+\frac{k^2(t)}{k^2(t)-\alpha^2}\left[-k_{\alpha2}\left(e_\alpha^2\right)^{\frac{p+1}{2}}-k_{\alpha3}\left(e_\alpha^2\right)^{\frac{q+1}{2}}\right]-l_{\alpha1}\tilde{\varepsilon}_\alpha^2\\ &-\frac{l_{\alpha2}}{1+p}\left(\tilde{\varepsilon}_\alpha^2\right)^{\frac{p+1}{2}}-\frac{l_{\alpha3}}{1+q}\left(\tilde{\varepsilon}_\alpha^2\right)^{\frac{q+1}{2}}+l_{\alpha1}\varepsilon_\alpha^2+\frac{2l_{\alpha2}}{1+p}\left(\varepsilon_\alpha^2\right)^{\frac{p+1}{2}}\\ &+\frac{2l_{\alpha3}}{1+q}\left(\varepsilon_\alpha^2\right)^{\frac{q+1}{2}}-k_{V1}e_V^2-\left(k_{h1}-\frac{1}{2}\right)e_h^2-\left(k_{\gamma1}-1\right)e_\gamma^2\\ &-\left(k_{\alpha1}-\frac{k^2(t)}{k^2(t)-\alpha^2}-\frac{1}{2}\right)e_\alpha^2-\left(k_{Q1}-\frac{k^2(t)}{2\left(k^2(t)-\alpha^2\right)}\right)e_Q^2\\ \leq&-k_1L-k_2L^{\frac{p+1}{2}}-k_3L^{\frac{q+1}{2}}+\eta\\ \leq&-k_2L^{\frac{p+1}{2}}-k_3L^{\frac{q+1}{2}}+\eta,\end{aligned}\tag{24}$$

where

$$
\begin{aligned}
k_1 = & \min\left\{2k_{V1}, 2k_{h1}-1, 2k_{\gamma 1}-2, k_{\alpha 1}\frac{k^2(t)-\alpha^2}{k^2(t)} - \frac{k^2(t)-\alpha^2}{2k^2(t)} - 1,\right.\\
& \left. 2k_{Q1} - \frac{k^2(t)}{k^2(t)-\alpha^2}, 2l_{j1}\right\},\\
k_2 = & \min\left\{2^{\frac{p+1}{2}}k_{j2}, \frac{l_{j2}}{1+p}2^{\frac{p+1}{2}}\right\},\\
k_3 = & \min\left\{2^{\frac{q+1}{2}}k_{j3}, \frac{l_{j3}}{1+p}2^{\frac{q+1}{2}}\right\},\\
\eta = & \sum_{j\in\{V,h,\gamma,\alpha,Q\}}\left[\frac{2l_{i2}}{1+p}\left(\varepsilon_i^2\right)^{\frac{p+1}{2}} + \frac{2l_{i3}}{1+q}\left(\varepsilon_i^2\right)^{\frac{q+1}{2}} + l_{i1}\varepsilon_i^2 + \kappa\sigma_i\varepsilon_i\right].
\end{aligned}
$$

Case 2: When $|e_\bullet| \leq \tau_\bullet$, the inequation (23) can be written as

$$
\begin{aligned}
\dot{L} \leq & \sum_{i\in\{V,h,\gamma,Q\}}\left[-l_{i1}\tilde{\varepsilon}_i^2 + \frac{2l_{i2}}{1+p}\left(\varepsilon_i^2\right)^{\frac{p+1}{2}} + \frac{2l_{i3}}{1+q}\left(\varepsilon_i^2\right)^{\frac{q+1}{2}} + l_{i1}\varepsilon_i^2\right.\\
& \left. + \kappa\sigma_i\varepsilon_i\right] - l_{\alpha 1}\tilde{\varepsilon}_\alpha^2 + l_{\alpha 1}\varepsilon_\alpha^2 + \frac{2l_{\alpha 2}}{1+p}\left(\varepsilon_\alpha^2\right)^{\frac{p+1}{2}} + \frac{2l_{\alpha 3}}{1+q}\left(\varepsilon_\alpha^2\right)^{\frac{q+1}{2}}\\
& - \left(k_{h1} - \frac{1}{2}\right)e_h^2 - \left(k_{\gamma 1} - 1\right)e_\gamma^2 - \left(k_{\alpha 1} - \frac{k^2(t)}{k^2(t)-\alpha^2} - \frac{1}{2}\right)e_\alpha^2\\
& - \left(k_{Q1} - \frac{k^2(t)}{2\left(k^2(t)-\alpha^2\right)}\right)e_Q^2 - k_{V1}e_V^2\\
\leq & -k_1L + \eta,
\end{aligned}
\tag{25}
$$

which ensures that the tracking errors and estimation errors of the closed-loop system can converge exponentially in the predefined compact set.

According to Lyapunov stability theory, all signals of the closed-loop system are semi-globally uniformly ultimately bounded. If $e_\alpha = k_a(t)$ or $e_\alpha = k_b(t)$, then $\frac{1}{k^2(t)-e_\alpha^2} = \infty$, which will cause the system instability. So, if the initial value of e_α is in the interval $(k_a(0), k_b(0))$, then it's going to stay within that interval. This completes the proof.
